# The “MYOCYTER” – Convert cellular and cardiac contractions into numbers with ImageJ

**DOI:** 10.1038/s41598-019-51676-x

**Published:** 2019-10-22

**Authors:** Tilman Grune, Christiane Ott, Steffen Häseli, Annika Höhn, Tobias Jung

**Affiliations:** 1 Department of Molecular Toxicology, German Institute of Human Nutrition Potsdam-Rehbruecke (DIfE), 14558 Nuthetal, Germany; 2 German Center for Diabetes Research (DZD), 85764 Muenchen-Neuherberg, Germany; 3 German Center for Cardiovascular Research (DZHK), 10117 Berlin, Germany; 4 NutriAct – Competence Cluster Nutrition Research Berlin-Potsdam, 14558 Nuthetal, Germany; 5 University of Potsdam, Institute of Nutrition, 14558 Nuthetal, Germany

**Keywords:** Software, Circulation, Microscopy, Software, Imaging

## Abstract

Measurement and quantification of cardiomyocyte or cardiac contractions as important (patho) physiologic parameters require highly specialized and expensive setups of fully integrated hard- and software that may be very difficult to use and may also depend on highly sophisticated methods of further data evaluation. With MYOCYTER (MC) we present a complete and highly customizable open-source macro for ImageJ, enabling fast, reliable user-friendly large scale analysis extracting an extensive amount of parameters from (even multiple) video recorded contracting cells or whole hearts, gained from a very competitive experimental setup. The extracted parameters enable extensive further (statistical) analysis to identify and quantify the effects of pathologic changes or drugs. Using videos following known mathematical functions, we were able to demonstrate the accuracy of MYOCYTER’s data extraction, also successfully applied the software to both cellular and animal models, introducing innovations like dynamic thresholding, automatic multi-cell recognition, “masked” evaluation and change of applied parameters even after evaluation.

## Introduction

In experimental cardiology, numeric quantification of cellular or cardiac contractions is essential for investigation of pathologic changes, aging or pharmacological effects both *in vivo* and *in vitro*. Such investigations are also of interest in scientific institutions, not specialized in cardiology and without access to the according highly integrated hard- and software solutions. Consequently, there is need for a solution including only standard imaging hardware and the free, user friendly MYOCYTER that is presented in this publication, enabling even beginners with basic knowledge to carry out such investigations and to extract a large variety of parameters in a fully automatized manner from experimentally obtained video files. MYOCYTER, a macro for the free image processing software “ImageJ”, has been already applied successfully to various different models *in vivo* and *in vitro* ranging from single cells to whole hearts.

One of the main and easiest existing applicable methods in this field is transmission light microscopy, used for the investigation of changes in the activity of contracting cells like cardiomyocytes. Firstly, MYOCYTER identifies cells by their movement, then, the two “primary” parameters velocity (“speed”, the difference between two subsequent images in a video) and amplitude (the difference between the current frame and a so-called “reference frame” that is either determined automatically or defined by the user) are extracted. The result is a sequence of greyscale images where differences are represented by intensity: no difference results in black, maximal difference in white pixels. “Contracting regions” are identified by summarizing the detected differences between the single frames, resulting in a single image representing the areas with the highest amount of movement during the whole video in brighter colors than areas with low or even without moving. After defining the according bright areas as “regions of interest” (ROIs), the contraction, i.e. the difference between the subsequent images (n) and (n + 1) (“speed”) within those areas and the difference to a reference frame (“amplitude”) is calculated and presented both as plot and numerical output that can be further statistically analyzed.

From both “speed” and “amplitude” extensive further information is calculated by MYOCYTER. Since cardiomyocyte contractions are investigated at framerates up to 1,000 frames per second (fps), very large stacks of images have to be analyzed per single cell and, consequently, for repeated experiments with large numbers of cells, data volumes will accumulate that are far beyond any manual evaluation. MYOCYTER offers a solution for the need for easy, user-defined and highly automatized methods of multi-cell mass analysis in the field of myocytic and cardiac (patho)physiology.

The free software “ImageJ”^1^ became a widespread tool for automatized large-scale image processing, that can be used to match very particular problems of analysis by recording and using individual “macros”^2–4^. Macros are instructions for ImageJ, written in a Java-like programming language. They are highly flexible tools, able to quickly process large amounts of files (both image and video) according to previously specified parameters^5^. Already available ImageJ plugins specialized on (cardio)myocytic evaluation are “FibrilTool”^6^, that quantifies the movement/contraction of fibrillary cell structures like microtubules or microfibrils, “SarcOptiM”^7^, using fast Fourier transform analysis of video frames as strategy for measurement of (changing) sarcomere length, and MUSCLEMOTION (MM), also quantifying cardiomyocytic contractions^8^. However, all these available macros lack several of the innovative features and parameters that are provided by MYOCYTER, like multi-cell recognition, dynamic thresholding, batch processing, and re-application of changed evaluation-parameters in real-time, need customized hardware, complex informatics or are highly specialized in only a single application^8,9^.

Aim of this study was the development of an easy to use, highly automated free software that enables the analyses of both (also multiple) spontaneously and electrically stimulated contracting single cells per video frame as well as whole muscle contractions by recording high-speed videos, extracting not only the mostly used parameters including amongst others systolic (used in this work corresponding to “contraction time”), diastolic (corresponding to “relaxation time”) and overall peak times for different customizable thresholds, and innovative methodological extensions, compared to the currently available software solutions.

MYOCYTER can be easily applied to videos provided by simplest light microscopy without any special features like rotating polarizers or additional equipment besides of a camera, recording >50 fps.

## Results

### Workflow and function of MYOCYTER

Use, functions and technical background of the macro MYOCYTER are described in detail in a separate manual (Online Supplement), as well as source code itself, commented and divided in its single functions for better overview and adaptability. A current version of MYOCYTER can also be found at www.scyrus.de (in the “ImageJ macros”-section) after publication of this work.

**Figure 1:**
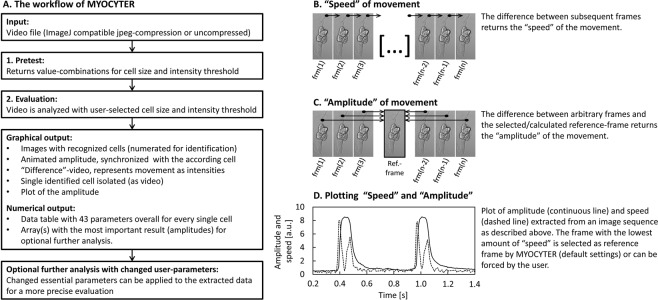
Workflow and basic functions of MYOCYTER. Panel A represents the typical evaluation of a video using MYOCYTER. Videos are first pre-tested (**1**.) to determine correct settings (for threshold and particle size) for videos containing multiple cells to be evaluated independently. This can also be skipped if the video contains only a single cell and if the entire frame is evaluated - but for a more precise evaluation with “masking” of the contracting object only, the pre-test is necessary. Subsequently, evaluation is performed (**2**.). MYOCYTER outputs a large number of different numerical and graphical results, which allow both quality control of the evaluation and a wide range of further statistics. Should a change of the applied user parameters be necessary, re-analysis in real-time is also possible. First, the two main parameters “speed” and “amplitude” are extracted. “Speed” represents the differences between the successive images of a video and indicates the speed of a movement: high speed correlates with high differences between consecutive pictures (see panel B). “Amplitude” represents the difference between every single frame and a “reference image” (either user-defined or calculated by MYOCYTER) (panel C). The reference image usually shows the examination object in its resting phase – thus, a high amplitude indicates a strong deviation from the resting state. Panel D shows both amplitude (continuous curve) and speed (dashed) for an isolated cardiomyocyte *in vitro*. Technically, speed can be considered as the absolute of the first derivative of the amplitude. From these two extracted values, MYOCYTER calculates 41 additional parameters.

Figure 1, panel A, provides an overview of MYOCYTER’s workflow when processing a video file. The data output is both graphically and numerically.

The macro determines the differences between successive images of video recordings (speed of contraction) (Fig. 1, panel B) as well as the differences between a user-defined or automatically selected reference image (usually from the resting phase of the cell/tissue) and all other images (amplitude of contraction) (Fig. 1, panel C), i.e. the scale of change compared to the resting state.

MYOCYTER is able to evaluate a video either “unmasked” or “masked”. “Unmasked” means here, the entire area of each video frame is evaluated and no distinction is made between various cells. This “unmasked” evaluation only makes sense if there is a single cell covering a large part of the video area. For a “masked” evaluation, a pre-test is necessary (Fig. 1, panel A), that allows to restrict the evaluation to the recognized moving structure(s) only.

This feature reduces massively the influence of static pixels or effects resulting from particles floating in the medium around the cell(s) of interest. If the cell area is small compared to the image area, the corresponding effects of static regions on amplitude and “speed” in the evaluation are quite significant (see below). A masking of the sample to be evaluated is, therefore, recommended.

The most important output of MYOCYTER is the numerical data in form of two files (“Results [date and time].txt” and “Amplitudes only [date and time].txt”) including all the results and applied settings from an evaluation, allowing complete retracement of the whole experiment^10–12^. “Results.txt” contains a large tabulator-separated data table for every single cell of every single video evaluated in the according file folder, listing 43 different parameters overall, additional statistics and the applied settings (Online Supplement, user guide). “Amplitudes only.txt” allows application of different parameters in real-time without re-evaluation of the whole video file.

**Figure 2:**
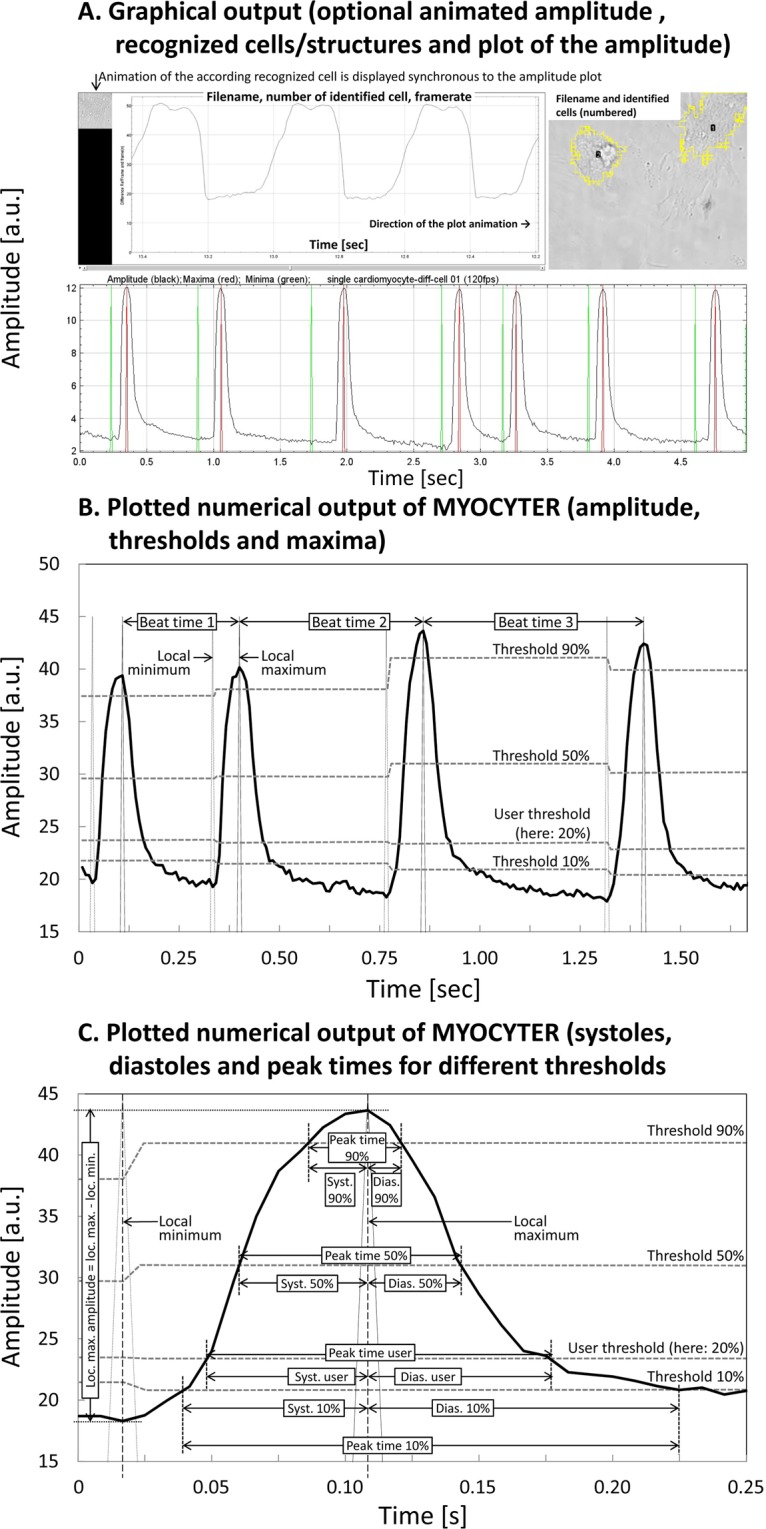
Extracted data, dynamic thresholding, systoles, diastoles and peak times. Panel A shows the main graphic output of MYOCYTER. Upper left image: optional animated output, the analyzed cell is animated synchronized to its amplitude. Identified structures (here, neonatal cardiomyocytes) are also indicated on a separate image (upper right), marked by a yellow outline and numbered individually. Furthermore, the amplitude of every single recognized cell/structure is plotted over time (bottom image). Panel B shows a few of the 43 extracted parameters (for details please see the manual, Online Supplement). The amplitude (thick black line) is “subdivided” by four thresholds (horizontal lines, light grey and dashed, representing the thresholds at 10, 20, 50, and 90%), that are re-calculated for every single recognized contraction. They represent the local minima + 10/20/50/90% of the difference between the local minima (dashed vertical spikes) and the next following maxima (continuous vertical spikes). Local (re)adjustment of dynamic thresholding enables “tracking” of even a shifted amplitude (thick black graph). The distance between two subsequent maxima determines the overall contraction time (“beat time”). If one of the thresholds is exceeded by the amplitude, timing of the corresponding individual systole begins. After the local maximum, timing of the corresponding diastole begins until the amplitude falls below the according threshold. Panel C of this illustration shows the four systolic, diastolic and overall peak times for every single threshold (horizontal light grey dashed lines, thresholds at 10/20/50/90%) which are calculated individually as % of the difference between local minimum (left vertical spike) and local maximum (right vertical spike), resulting in “Local maximal amplitude”.

Furthermore, there is a graphical output which indicates moving structures or cells recognized in the video, the plots of the corresponding amplitudes including recognized maxima and minima, as well as an (optional) video output showing each identified structure or cell synchronized with its corresponding amplitude (Fig. 2, panel A).

Images and videos can be used for presentation and offer a brief note of the applied settings - for more details please see the manual (Online Supplement, user guide).

Detailed analysis and comparison of samples/groups depend on the numerical output. The 43 extracted parameters enable a detailed statistical evaluation and comparison of cells under different treatments or disease states. Movement of contracting cells is described via a large variety of parameters (frame number, absolute time, amplitude, recognized local maxima and minima, speed, elapsed time since last contraction, previously counted contractions, four different thresholds and the according systoles, diastoles and overall peak times as depicted in Fig. 2, panels B,C). At least one of those should differ from each other, if cells affected by different treatments/pathologic changes are compared^13,14^.

### Validation of MYOCYTER’s accuracy and reliability by defined and known image sequences

To test MYOCYTER’s validity, videos with defined and known content were used. The results for amplitude and speed of the evaluation corresponded reproducibly to the exact expected values.

#### A sequence of inverting images

**Figure 3:**
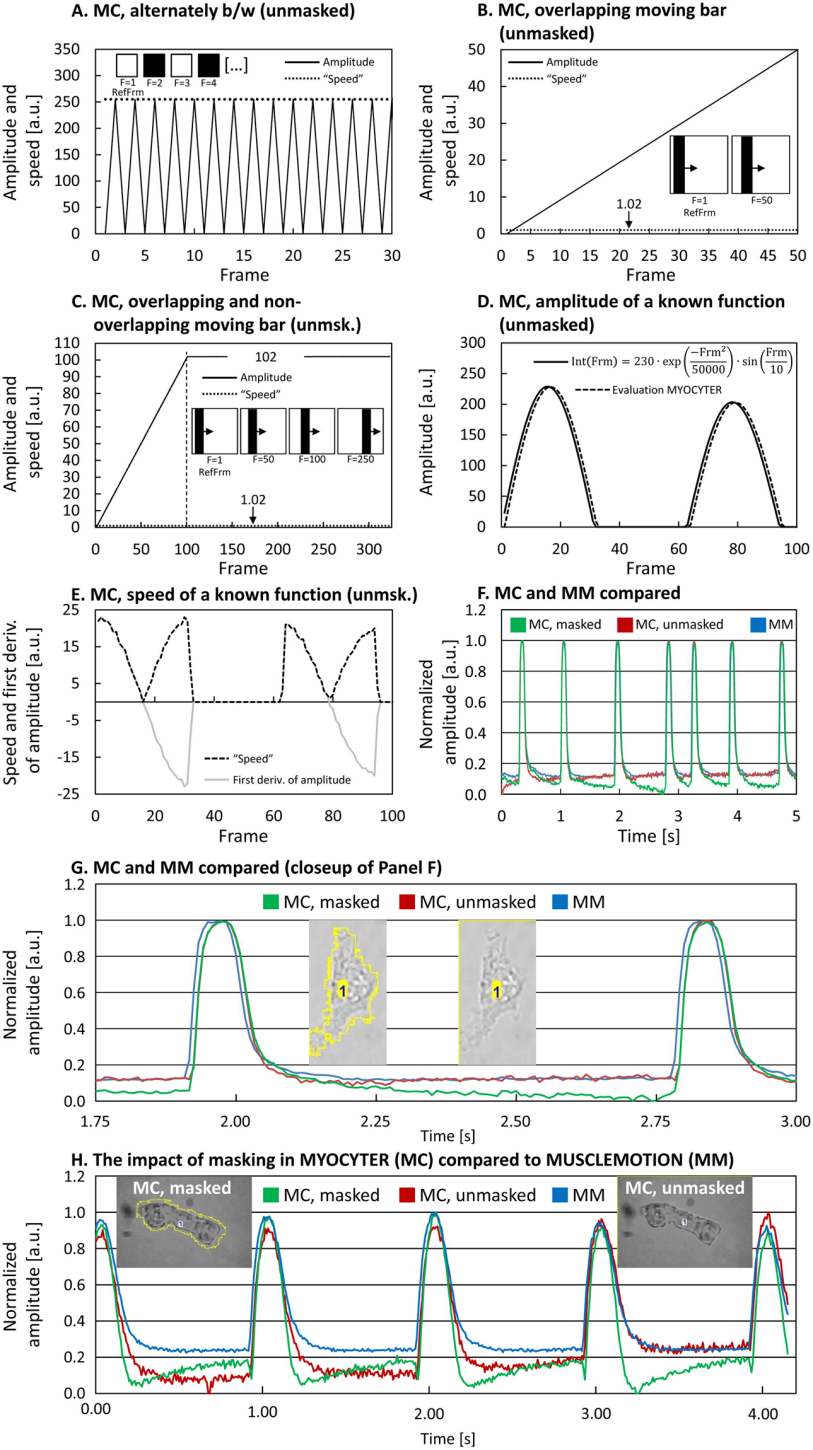
Validation of MYOCYTER (MC) and comparison with MUSCLEMOTION (MM). To validate the reliability of MYOCYTER’s (MC) output, videos with known parameters were analyzed and the macro was also compared to the recently published MUSCLEMOTION (MM). Panel A: Analysis of a video inverting from frame to frame returned the expected values for speed (constant at 255) and amplitude (change of 255 from frame to frame). Video: “alternating bw.avi”, Settings (A-E): LowerThreshold = 0; RefFrame = 1. Panel B: Analysis of a black bar moving from left to right on a white background. Amplitude (solid line, difference between reference frame and current frame) increases absolutely linearly. Here, first frame was defined as reference. “Speed” (dotted line) indicates the difference between subsequent images. Video: “moving bar.avi”; Panel C: Same video as in panel B, but in the range from frame 1 to frame 325. After frame 100, the black bar no longer overlaps with its position in the reference image (frame 1), so the change in its linear motion tends linearly towards a limit (reached after 100 frames). Panel D,E: Analysis of a video following a known equation (“declining function.avi”, Online Supplement). Panel F–H: Evaluation of a cardiomyocyte *in vitro* with different macros (MYOCYTER, MC and MUSCLEMOTION, MM). “Masked” indicates restriction to the recognized moving structure only (MC, green curves), “unmasked” evaluation with MC (red curves) and evaluation with MM (blue curves). Microscopic images represent the according quantified cells, yellow outlines of cells indicate “masked” evaluation (restricted to outlined area, only available in MC). Settings (F, G): Lower Threshold = 77, Cellsize = 515; Settings (H): Lower Threshold = 66, Cellsize = 2067.

A video composed of inverting images (alternating black and white) is expected to return a constant difference of 255 from frame to frame (the maximum possible change in 8-bit grayscale) as speed and an amplitude that changes between 255 and 0 from frame to frame. Every second frame is identical to the defined reference frame (in this case the white frame 1), resulting in an amplitude of zero, while the other frames (black) show maximum difference (255), as depicted in Fig. 3, panel A.

#### Uniformly moving structures

The first test video (no compression applied) depicts a black bar moving (for the first 100 frames overlapping) from left to right on a white background. The area of the video is 500·500, the bar is 100·500 pixels, moving exactly one pixel per frame for an overall of 50 frames, thus 500 pixels are inverting from image to image both on front and back of the bar, summarizing a total of 1000 inverting pixels (Fig. 3, panel B). The first image of the film was defined as reference frame and the amplitude returns the expected deviation from this reference image for each individual frame (Fig. 3, panel B, sold line). “Speed” (Fig. 3, panel B, dotted line) indicates the difference between one image and the next one, in this case constant at a value of 1.02 (averaged over all 50 frames). The theoretically expected value for “speed” is (1000 changing pixel/250,000 pixel overall)·255 = 1.02, thus, no derivation is detectable. By the same value (1.02), the amplitude should increase from image to image. MYOCYTER returned an average of 1.02 (over all 50 frames), also corresponding to no measurable difference.

Evaluation of the same video file for an overall of 325 frames is depicted in Fig. 3, panel C. The amplitude increases strictly linearly up to frame 100, remaining constantly at 102 (mean of frame 101 to frame 325). After frame 100, the black bar no longer overlaps with its position in the reference image (frame 1), so the change in its linear motion tends linearly towards a limit (first 100 frames). Compared to the reference image (after frame 100 the maximum possible change is achieved), an area of 100·500 pixels changes from white to black, and another from black to white, so 100,000 pixels are inverted of a total area of 250,000 pixels. The theoretically expected value for the maximal amplitude is therefore (100,000/250,000)·255 = 102, exactly matching MYOCYTER’s output. The slope of the amplitude in the linear range up to frame 100 is 1.02 (mean) and shows no deviation from expectation.

Though, using a compressed video file (MJPEG compression, see Online Supplement), slight differences can be detected: the values for speed change to 1.049 ± 0.073285 (averaged, panel B), and 1.041 ± 0.029231 (averaged, panel C), while the amplitude after frame 101 is found at 102.459 ± 0.370569 (averaged, panel C), slightly differing from the expected values 1.02 and 102, respectively. Those differences seem to be a result of lossy data compression.

#### Sequences of changing pixel intensities

Analyzed was a video of known intensity for every individual frame. The intensity (8-bit grayscale) follows a given equation (Fig. 3, panel D, solid line, a decaying sine function, values less than zero are defined as zero, the equation is shown in the panel). This function starts at frame = 1 and is therefore not 0 but 22.9612 (result of the equation). Since 8-bit intensities have to be integers between 0 and 255, the results were rounded down internally by ImageJ (that was used for creation of this video) via the function “floor(number)”, thus, 22.9612 was rounded down to 22. Due to this forced rounding, no differences after the decimal point were detectable and MYOCYTER returned the exact integers of the whole function without any fail (Fig. 3, panel D), but shifted for one frame, because the amplitude is the difference between the current and the reference frame, thus MYOCYTER starts with a zero (frame 1 was defined as reference frame). Both speed (Fig. 3, panel E, dashed black line, equals the absolute value of the first derivative of the amplitude) and the first derivative of the amplitude (continuous grey line, calculated from the amplitude itself as difference between amplitude of frame [n] and [n + 1]) superimposed perfectly (same video analyzed as in Panel D).

#### MYOCYTER’s “masking”-feature compared to other software (MUSCLEMOTION)

Evaluation of a cardiomyocyte (“single cardiomyocyte.avi”, Online Supplement) *in vitro* using both MYOCYTER (MC) and MUSCLEMOTION (MM, another ImageJ macro which could resist the gold standards in such evaluations, providing similar but more limited functions, extracts only amplitude and speed as numeric parameters^8^) is depicted in Fig. 3, panel F,G. The red graph represents evaluation using MC in “unmasked”-mode (analysis is not restricted to the recognized cell only). The result of MC (“unmasked”) is virtually identic to the result of MM (blue curve, MM includes no “masking”-function), that also evaluates the whole frame. The green graph represents evaluation of MC (“masked”-mode) using its results from the pre-test. Figure 3, panel G shows a section of panel F including the analyzed parts of the according video file: the left image represents “masked” analysis (only the yellow outlined area is analyzed) by MC (green curve), while the right one represents the “unmasked” one by both MC (red) and MM (blue) analyzing the whole image. Masking of the cell in MYOCYTER significantly attenuates the extracted amplitude (Fig. 3, panels F, G, green curve).

Figure 3, panel G depicts analysis of an adult cardiomyocyte (“adult cardiomyocyte small.avi”, Online Supplement). Here, the area of the cell is small compared to the whole image area (right microscopic image). Restricting the analysis to the cardiomyocyte only (left image) shows even more impact on the result than in Fig. 3, panels F, G where the cell is larger compared to the whole image. Evaluation by MM (blue curve) and by MC in “unmasked” mode (red) lose details of the contraction, that may contain important quantifiable information (pathologic changes or effects of drugs), compared to “masked” analysis with MC (green).

However, “unmasked” evaluation includes also pixels (noise, floating bubbles or cellular fragments) not involved in the cellular contraction that may cause significant distortion of the results, depending on the proportion of the moving structure in the overall picture.

In contrast, using MYOCYTER’s “masked” evaluation (in this case, the pretest is necessary), analysis is restricted only to the detected cell(s), which clearly minimizes those disturbances, improving precision and comparability of the results.

A detailed comparison of the technical aspects and performance of MYOCYTER and MUSCLEMOTION can be found as table in the Online Supplement.

#### Dynamic thresholding of a shifting amplitude

Both amplitude and speed are differences between individual frames of a video, not only impacted for example by contractions of a cardiomyocyte, but also by camera noise, uneven or changing illumination, organelles passively moving inside even a dead cell (Brownian motion), by cell debris or bubbles floating into the field of view, as well as by vibrations of the setup.

**Figure 4:**
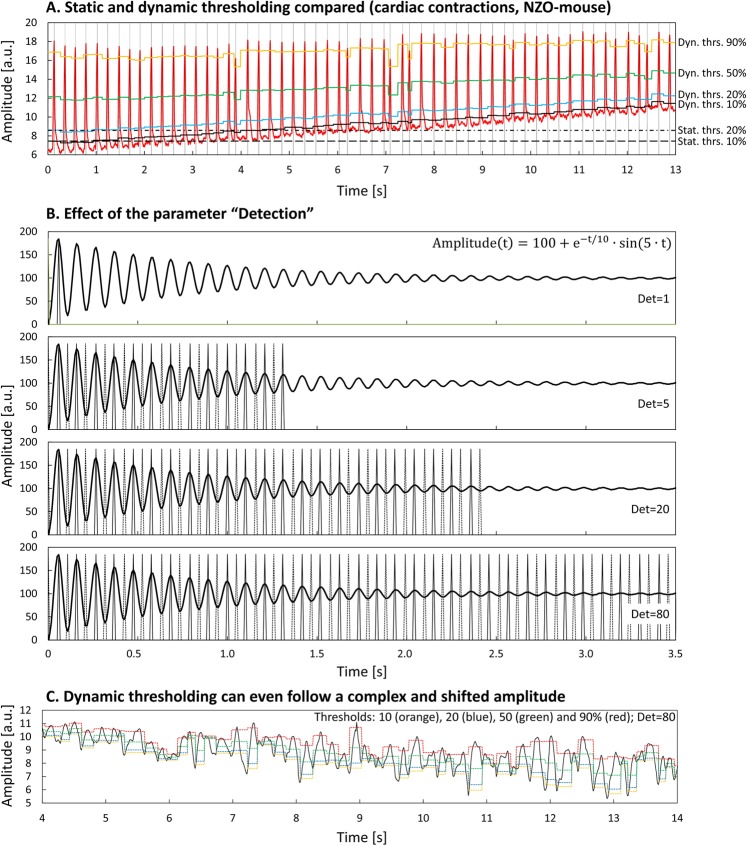
MYOCYTER’s dynamic thresholding and peak recognition. Panel A: Difference between static and dynamic thresholding. A shifted amplitude (red curve) of cardiac contractions is tracked easily by dynamic thresholding, able to adapt to the entire curve also including local maxima of different height. Horizontal solid lines represent the corresponding dynamic thresholds at 10 (black), 20 (blue), 50 (green), and 90% (orange) of the local peak height. Static thresholds, defined at the beginning of the video (dashed horizontal line: at 10%, dots and strokes: at 20% of the first maximum), failed and “lost” the amplitude completely after a short time (static threshold at 10% after 4 seconds, static threshold at 20% after 7 seconds). Vertical dashed lines indicate detected local maxima. Panel B: Influence of the parameter “Detection” on recognition of maxima (continuous vertical spikes) and minima (dotted vertical spikes). The amplitude (thick black curve) is also given by a known time-dependent function (see top figure of this panel). “Detection” determines how large the deviations of local maxima and minima may be from each other to be recognized as such. With higher values for “Detection” (here, values of 1, 5, 20, and 80 are applied) smaller differences are recognized as local maxima and minima. Panel C: MYOCYTER is even able to track a complex, irregular and shifted amplitude (black continuous graph). Applied values for the four thresholds (horizontal dashed lines) and “detection” are shown in the panel. For a clearer presentation, maxima and minima are not displayed here.

In practical application, differences between frames of a video are thus practically always above zero. These differences, which may accumulate during measurement, may not be caused solely by the moving object under examination, and may induce a shift of the amplitude along the y-axis over time. However, this shift can be successfully balanced by “dynamic thresholding”, which adjusts the four different thresholds after each detected minimum to the immediately following maximum, thus enabling precise tracking of even a shifted amplitude, while static/constant thresholds would fail completely. In order to compare static and dynamic thresholding, the cardiac contractions of a NZO-mouse (heart surgically exposed, filmed directly at 240 fps) were analyzed (Fig. 4, panel A): the amplitude (red curve) shifted slightly over time, but dynamic thresholding (at 10 (black), 20 (blue), 50 (green), and 90% (orange) of the local peak height) was able to precisely adapt to the graph, while static thresholds, defined at the beginning of the video, completely failed after a few seconds.

#### Changing amplitudes and *arrhythmia*

While the contraction amplitude of cells or a heart under normal conditions does only change very slightly during measurement, certain treatments or pathological conditions like arrhythmia may result in massive differences between local amplitudes during video recording - also here, dynamic thresholding turned out to be of advantage. The most important parameter for the user influencing correct detection of maxima and minima is the setting of “Detection” (for details please see the manual in the Online Supplement). Higher values of “Detection” provide a more sensitive identification of maxima and minima. For cardiomyocytes *in vitro*, values in the range of 5–10 provide a good recognition. Nevertheless, in cases where the (local) amplitudes from contraction to contraction are very different, small maxima may not be recognized, if the value for “Detection” is set to low.

Recognition of maxima with very different amplitudes was examined using a video whose image intensities follow a known function (Fig. 4, panel B): increasing “Detection” finally up to 80, results in perfect recognition of all local peaks and valleys. Though, further increase will finally end up in recognition of even noise as maxima and minima. The proper value can be estimated by the global highest and lowest value of the whole plot: if the user wants to detect peaks of about 20% of this range, a “Detection” of about 5 should be applied (detection ≈ inversed share of the maximal amplitude to be detected).

If the value for “Detection” was not applied perfectly in the first evaluation, it is *not necessary* to re-analyze the whole material again (please see the manual, Online Supplement).

After setting an appropriate value for detection, MYOCYTER is able to accurately follow even both complex and shifted amplitudes, which show very significant deviations in local maxima as depicted in Fig. 4, panel C: even a complex, irregular and shifted amplitude of a neonatal cardiomyocyte isolated from a C57BL/6 mouse (unpaced measurement) with significant differences in the local amplitudes, as well as a shift of the amplitude (black continuous graph) over time tracked perfectly.

Thus, MYOCYTER is even able to evaluate “difficult” data and also compensates for changes in illumination during measurement (a typical cause for shifted amplitudes).

#### Impact of thapsigargin on isolated cardiomyocytes (C57BL/6J mouse)

**Figure 5:**
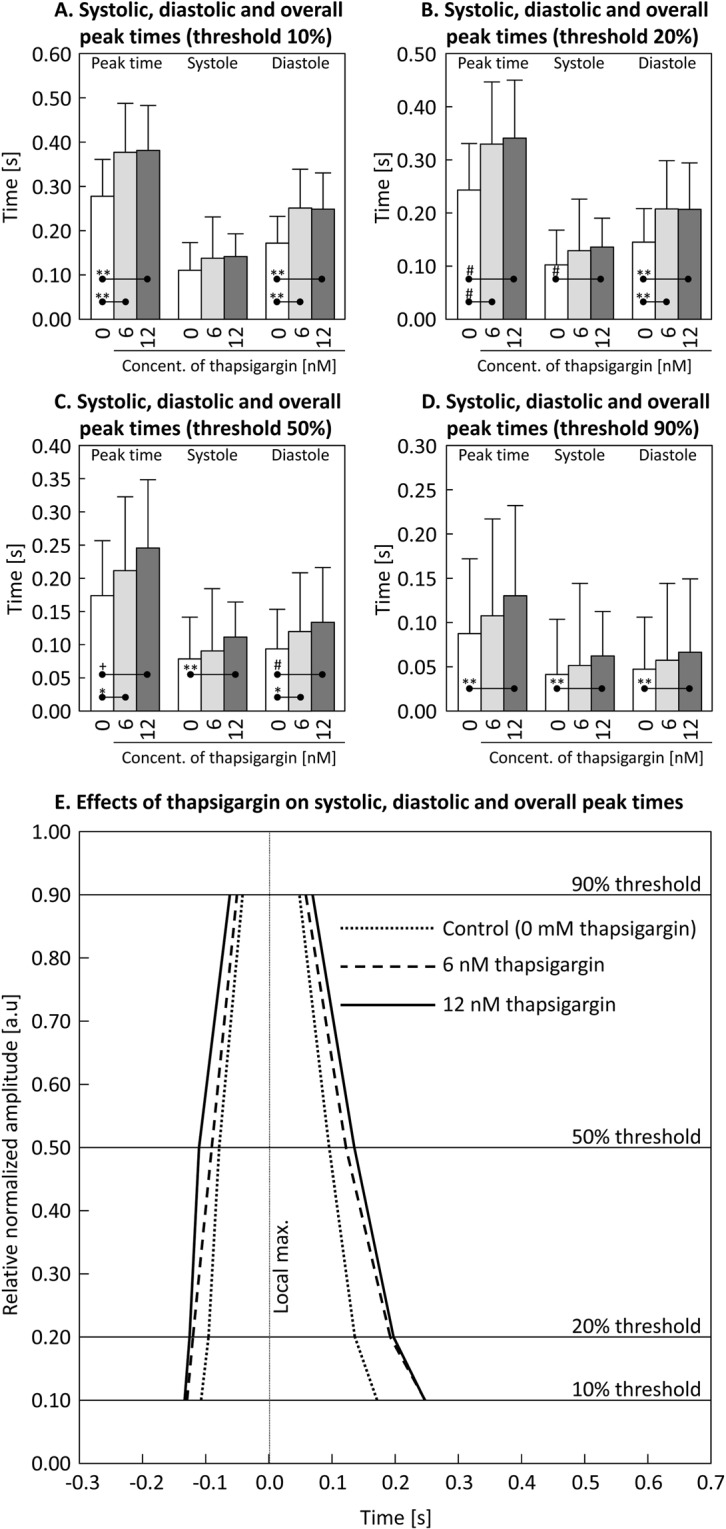
Impact of thapsigargin on isolated C57BL/6J-mouse cardiomyocytes. In this figure, the impact of different thapsigargin concentrations on the overall peak times, systoles and diastoles of isolated adult C57BL/6J-cardiomyocytes is depicted (paced measurement). The different panels present the results measured at four different thresholds: 10 (Panel A), 20 (Panel B), 50 (Panel C) and 90% (Panel D). White columns (“0”) indicate the untreated control, “6” (light grey columns) and “12” (dark grey columns), represent the according nanomolar concentrations of thapsigargin applied. The (overall) peak time is the sum of both its systolic and diastolic share of the according threshold. Panel E displays the normalized amplitude of a single contraction for untreated control cells (dotted black line) compared to cells exposed to 6 nM (dashed black line) and 12 nM (continuous black line) thapsigargin, respectively. The four thresholds (10, 20, 50, and 90%) are represented by accordingly labelled horizontal lines, while the vertical dotted line represents the detected local maximum. Statistics: significant differences are indicated by connecting lines; *(p < 0.05), **(p < 0.01), ^#^(p < 0.005), and ^+^(p < 0.001); one-way ANOVA.

To demonstrate the ability of MYOCYTER to not only measure contractions using various thresholds, but also to distinguish it into systolic and diastolic part, isolated adult C57BL/6 J mouse cardiomyocytes were exposed to different concentrations of thapsigargin (blocker of sarcoplasmic reticulum Ca^2+^-ATPase) (Fig. 5).

As the concentration of thapsigargin increases, both the total peak time of a contraction increases (for the thresholds at 10, 20, 50, and 90%), as well as the duration of the corresponding systoles and diastoles, as shown in Fig. 5, panel A–D. The individual changes by thapsigargin are depicted in panel E, whereby the duration of the diastolic phase increases significantly more than the systolic phase in a concentration dependent manner.

#### The influence of EtOH on various cardiac contraction parameters of Daphnia pulex

Please see Online Supplement for the according results.

#### Online content

The source code of MYOCYTER, a very detailed manual as well as several example videos are available as online content.

## Discussion

Summarizing the results, with MYOCYTER a powerful and versatile tool is provided. Not only is it superior to the existing MUSCLEMOTION, it provides also higher precision via “masked” evaluation (Fig. 3, panel F-H), extracts many more parameters (please see the user manual, Online Supplement) that describe cellular contractions in unmatched detail, allowing a much more advanced statistical analysis, and furthermore enables a very convenient experimental setup. The high utility of these parameters was proven investigating the effects of thapsigargin on cardiomyocytic systolic and diastolic share, the reliability of MYOCYTER was proven using known videos (Fig. 3, panels A–E) and it’s versatility by investigation of different models from isolated single cells to whole hearts *in situ* (Fig. 4, panel A,C, Fig. 5, Fig. Supplement 1).

Nevertheless, the evaluation with MYOCYTER has certain limits that should be considered and that are also discussed in the following.

The amplitude is given in “arbitrary units” and should be used accordingly “carefully” in the statistical comparison of experimental groups. We were able to show that the amplitude (in case of “unmasked” evaluation) is influenced among other things by the area ratio of the cell to the overall image (Fig. 3, panel F–H). Comparing the amplitudes of different cells from different groups is therefore less reliable than the comparison of absolute values (expressed in seconds such as systole, diastole, total peak time and the interval between two contractions). A comparison of the amplitudes is, however, useful when examining the same cell before, during and after a treatment. Also, the shape of amplitudes (in detail “described” by the data delivered via the four thresholds) can be used to compare different groups affected by pathologies or drugs.

Comparing the amplitudes extracted from the same video files (see Online Supplement), MYOCYTER virtually produces the same amplitudes as recently published software tools, e.g. MUSCLEMOTION (Fig. 3, panels F-H) that was compared successfully to different existing gold standards (optical flow, post deflection, edge detection systems and manual analysis)^8^.

However, MYOCYTER returns not only better graphical output, like a video output of the measured cells synchronized with their contractions (for error checking, Fig. 2, panel A), is able to recognize and analyze several cells in the same video independently (Fig. 2, panel A) and allows also further data processing with different parameters applied for thresholds and “Detection” (a parameter defining the sensitivity with which maxima and minima are detected) in real time, without re-evaluation of the video. In the sum, MYOCYTER was proven to be the ideal choice.

Absolute units, on the other hand, are ideal for comparison of different samples. Many treatments or pathologies influence parameters like systole, diastole, total peak time, and the distance between two contractions, which can be statistically compared with each other as absolute values in seconds according to Fig. 2, panel C, enabling detection of corresponding effects. In this case, seconds may be more reliable than the arbitrary values of the amplitude, since the amplitude also depends on characteristics like the “transparency” of a cell that impacts the difference recognized between subsequent images, even if two cells show the same amount of movement. This “transparency” can also be affected by the amount of visible organelles within otherwise identical cells, resulting in different amplitudes.

**Figure 6:**
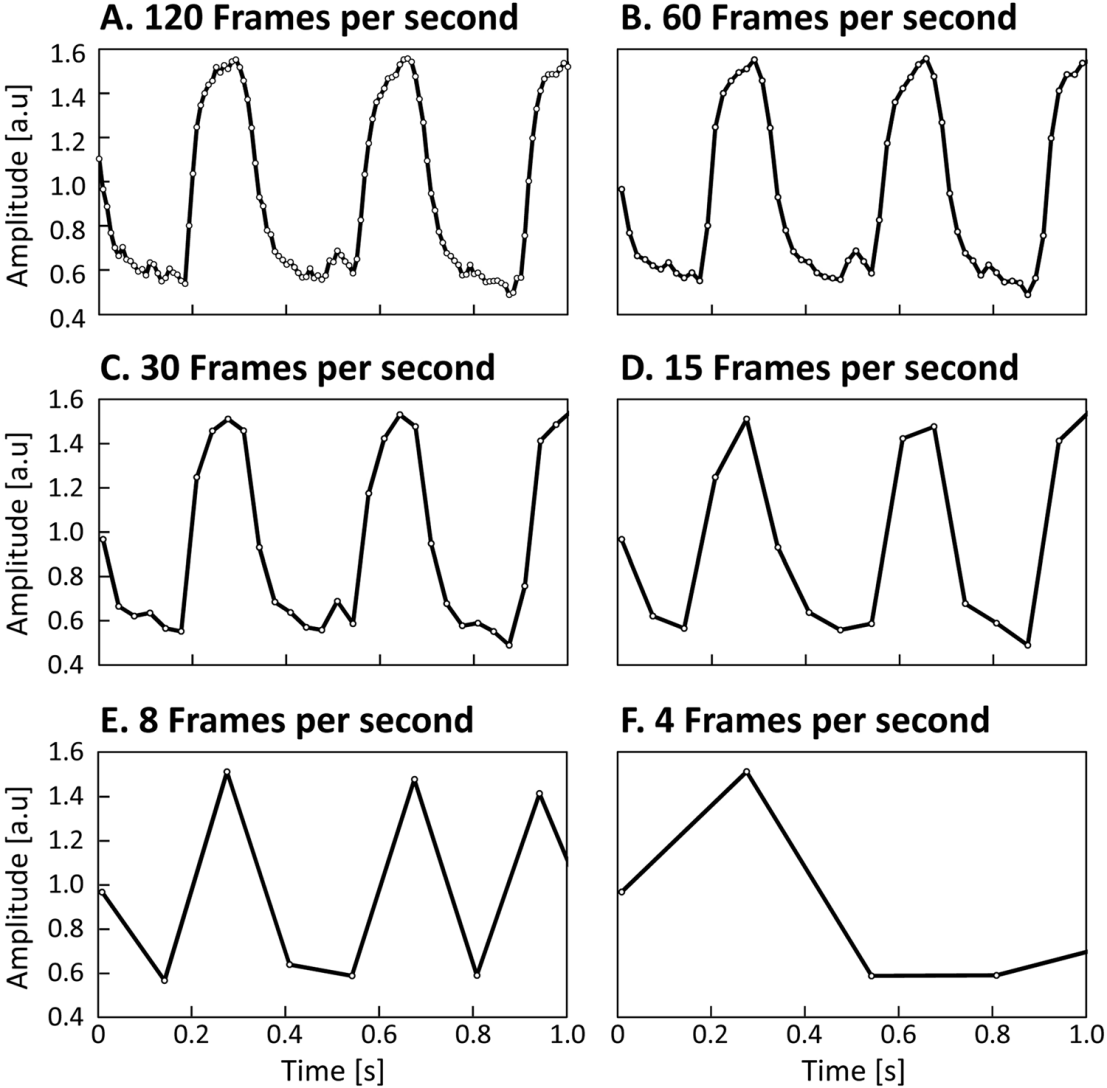
Influence of the frame rate on the amplitude during evaluation with MYOCYTER. This figure shows the effects of the frame rate on the final plot of the amplitude in an evaluation with MYOCYTER. A representative cardiomyocyte (isolated from a C57BL/6J-mouse heart) contracts about 3 times per second, so at 120 fps there are about 40 data points/frames per contraction available (panel A). If the frame rate of the video is reduced (halving from panel B–F), correspondingly fewer measuring points are available describing the shape of a contraction. The shape of the curve loses detail until a complete contraction is only represented by a single measurement point (panel E). In panel F, the frame rate is so low that the information of complete contractions is missing.

An appropriate frame rate is the most important requirement for a detailed representation of the shape of a contraction. While for isolated cardiomyocytes, frame rates of ≥100 fps provide sufficient measurement points per contraction (in our example, about 40, see Fig. 6, panel A), this would no longer be the case *in situ* when filming the heart of *Suncus etruscus* (Etruscan shrew) for example. Shrew hearts contract up to 20 times per second^15^, so technically only 5 measurement points remain at a rate of 100 fps to describe a whole contraction (Fig. 6, Panel D–F). Such few measuring points are not nearly sufficient for a detailed representation of systole and diastole as shown in Fig. 2, Panel C. Statistical comparison of different groups based on these parameters would no longer be possible, or would require frame rates of at least 1000 fps. About 40 measurement points per single contraction should therefore be regarded as the lowest limit for reliable statistics.

Dynamic thresholding proved to enable dynamic adaption (Fig. 4, panel A) of the evaluation even to extremely complex and irregular amplitudes (Fig. 4, panel C), that may result from arrhythmic contractions paired with changes of background illumination during measurement, that is also interpreted as increasing difference between the current and the reference frame. Consequently, this innovation furthermore reduces the requirements for the experimental setup, without compromising evaluation quality itself.

The impact of pathologies or pharmaceutical effects on cardiomyocytic or heart function is also an important focus of this field. In our study, the impact of frequently used agents on cardiomyocytes (thapsigargin) and hearts (ethanol) was investigated. Both agents revealed clear effects that were detectable using MYOCYTER.

Thapsigargin is a potent, cell-permeable agent that releases Ca^2+^ via inhibition of the endo-/sarcoplasmic reticular Ca^2+^-ATPase. According to Sankaranarayanan *et al*., thapsigargin decreases systolic and increases diastolic intracellular Ca^2+^ ^16^, resulting in both increase of systolic and diastolic times. The sarcoplasmic reticulum controls the amount of Ca^2+^ released into the cytosol and at the same time the force development during the systole, while remove of the Ca^2+^ depends on action of the Ca^2+^-ATPase, determining diastolic speed and amplitude^17^. Also, the increased concentration of cytosolic Ca^2+^ affects the release of Ca^2+^ during the systolic part of a contraction, thus both systolic and diastolic times increase as shown in isolated rabbit hearts^16^. Here, we were able to reveal this effect in cardiomyocytes (Fig. 5). Consequently, MYOCYTER enables easy and reliable detection of effects caused by widely used agents in cardiology like thapsigargin or others that impact cytosolic Ca^2+^-regulation and at the same time systolic and diastolic intervals.

Investigation of the impact of ethanol on the hearts of *Daphnia pulex* (*in situ*, see Online Supplement) revealed at lower alcohol concentrations an increase in systolic time, while the according diastole was shortened – in the sum, total peak time increased. At higher concentrations, higher increase in diastolic compared to systolic time was found. Consequently, using MYOCYTER we were able to quantify not only changes of overall contraction time, but also their systolic and diastolic shares.

In addition to the biological one, the macro may also be interesting for technical applications.

## Methods

### Microscopy and recording

Samples were investigated with a *Zeiss* “LSM780” confocal laser scanning microscope at 10- (water flea hearts) or 20-fold (single cells) magnification, respectively, in transmission light mode. Videos were recorded at framerates of 120 or 240 fps, using different middle class smartphones (including iPhone6s and different Samsung Galaxy models) attached to the ocular using an adapter (Vizzlema “*Universal*”)^18,19^. Exposed mouse hearts were filmed directly with an iPhone6s.

### Image processing and analysis

To run MYOCYTER, a current version of ImageJ (version 1.52a) is highly recommended. Running the macro in FIJI (the recursive acronym for “*Fiji Is Just ImageJ*”, also an image processing package based on ImageJ) may end up with an error message, especially since in FIJI after some updates the naming of open windows has changed and images or videos therefore may be no longer recognized correctly. Though, the current version of MYOCYTER works perfectly with FIJI version 2.0.0-rc-69/1.52n.

### NZO/HIBomDife and C57BL/6J mice

The animals were housed at 22 °C with a 12 h light-dark cycle (lights on at 6.00 a.m.) in type II Makrolon cages. Wooden gnawing sticks avoided excessive teeth growth. The mice were kept according the NIH guidelines for care and use of laboratory animals, also all experiments were approved by the Ethics Committee of the State Ministry of Agriculture, Nutrition and Forestry (State of Brandenburg, Germany)^20^.

### Recording whole heart contractions of an NZO mouse

Male NZO mice (9.5 months old, NZO/HIBomDife, German Institute of Human Nutrition Potsdam-Rehbruecke [DIfE], Nuthetal, Germany) were housed under (12 h light/dark cycle, 21 °C room temperature, standard diet and free access to food and water). For imaging, a whole NZO mouse heart and isolation of adult cardiomyocytes, mice were anesthetized (isoflurane 3–5%) prior to cervical dislocation. Mice chests were opened to expose the still beating heart, immediately used for recording whole heart contractions.

### Isolation of cardiomyocytes from neonatal mice

1–3 days old neonatal C57BL/6J mice were decapitated using sterile scissors and chest was opened to extract the heart. Isolation of cardiomyocytes was performed using Pierce™ Primary Cardiomyocyte Isolation Kit (88281) according to the manufactures instructions. In contrast to cardiomyocytes isolated from adult animals, these cells were not electrically stimulated during measurement (“unpaced measurement”).

### Isolation of cardiomyocytes from adult mice

Male C57BL/6J mice (4 months old; Janvier Labs, Le Genest-Saint-Isle, France) were housed under identical conditions as the NZO mice. Mice were anesthetized (isoflurane 3–5%) prior to cervical dislocation, heart was removed and cardiomyocytes were isolated according to Ackers-Johnson *et al*.^21^ Isolated cardiomyocytes were kept in a Krebs-Ringer bicarbonate buffer (137 mM NaCl, 5.4 mM KCl, 0.5 mM MgSO_4_-heptahydrate, 10 mM D-glucose, 1 mM CaCl_2_-dihydrate, K_2_HPO_4_-trihydrate, 25 mM NaHCO_3_) - modified according to Heinzel *et al*.^22^ - without 2,3-butanedione monoxime (BDM) (*Sigma*, B0753) and immediately transferred onto a glass cover slip, usable for the sample chamber system and an inverted motic fluorescence microscope from IonOptix. After 10 sec settling time, cells were acutely stimulated (pacing frequency of 1 Hz, 4.6 ms square pulses, 10 V, 37 °C), by a pair of platinum electrodes in the chamber connected to an electric field stimulator (“Myopacer”, from *Ionoptix*). Thus, recording of adult cardiomyocyte contractility was performed under controlled pacing conditions (“paced measurement”). Thapsigargin, a potent, selective, cell-permeable and irreversible blocker of sarcoplasmic reticulum Ca^2+^-ATPase (from *Sigma*, T9033, 1 mg/ml in DMSO, 1.536 mM) was diluted in buffer (see above) to a final concentration of 6 and 12 nM, respectively and isolated cardiomyocytes were pre-incubated for 30 minutes.

### Water flea (*Daphnia pulex*)

The water fleas were used as easy example of whole animal cardiac contraction and obtained from an aquarium retailer who supplied them in a nutrient solution. Alcohol was pre-diluted in nutrient solution given directly to the animals until the appropriate concentration (0.1, 0.3, 0.5, and 2.0% v/v, respectively) was reached. The animals were incubated for 20 minutes and then measured as quickly as possible: 5–6 animals were simultaneously placed in nutrient solution with a pipette on a microscope slide and fixed with a coverslip. Each animal heart was filmed for about 10 seconds at 120 fps.

### Statistics

The numerical data obtained from MYOCYTER were further processed in *GraphPad*’s “Prism” (v.6.07) via two-tailed Student’s t-test or one-way ANOVA (Tukey correction applied). A p < 0.05 was considered to be statistically significant. p-Values are indicated in the according figures.

## Supplementary information

Additional Results

Manual for MYOCYTER

MYOCYTER macro code for ImageJ

MYOCYTER macro code for ImageJ (zipped)

Small adult cardiomyocyte

Alternating frames (b/w)

Adult cardiomyocyte

Declining function (intensity)

Declining function (intensity) uncompressed

Exposed mouseheart

Moving bar

Single cardiomyocyte

Two cardiomyocytes

Water flea heart
