# Supplementary material for: The “MYOCYTER” – Convert cellular and cardiac contractions into numbers with ImageJ
Source: Sci Rep. 2019 Oct 22;9:15112. doi: 10.1038/s41598-019-51676-x (PMC6805901; doi:10.1038/s41598-019-51676-x)
Supplement: Supplementary file 1 — Additional Results [file 41598_2019_51676_MOESM1_ESM.pdf]

Online Supplement

The “MYOCYTER” –
Convert cellular and cardiac contractions into numbers with ImageJ

MYOCYTER – An analysis tool to convert cellular and muscle tissue contractions into numerical and graphical data with ImageJ

Tilman Grune[1,2,3,4,5] MD, Christiane Ott[1,3] PhD, Steffen Häseli[1] B.Sc, Annika Höhn[1,2] PhD, and Tobias Jung[1,3] PhD

[1] Department of Molecular Toxicology, German Institute of Human Nutrition Potsdam-Rehbruecke (DIfE), 14558 Nuthetal, Germany
[2] German Center for Diabetes Research (DZD), 85764 Muenchen-Neuherberg, Germany
[3] German Center for Cardiovascular Research (DZHK), 10117 Berlin, Germany
[4] NutriAct – Competence Cluster Nutrition Research Berlin-Potsdam, 14558 Nuthetal, Germany
[5] University of Potsdam, Institute of Nutrition, 14588 Nuthetal, Germany

Correspondence: Tobias Jung, Department of Molecular Toxicology, German Institute of Human Nutrition Potsdam-Rehbruecke (DIfE), Arthur-Scheunert-Allee 114-116, 14558 Nuthetal, Germany; e-mail: tobias.jung@dife.de; phone: +49 (0)33200 88-2490

Results

The technical aspects and performance of MYOCYTER and MUSCLEMOTION compared

The technical innovations provided by MYOCYTER include:

- **Automatic recognition of multiple contracting structures:** Multiple cells in the same video are recognized and evaluated independently. In contrast, MUSCLEMOTION is only able to evaluate videos with a single contracting structure. Our feature saves experimental time, because several cells can be recorded at the same time and later be evaluated independently without the need for additional manual processing of the videos.

- **"Masked" evaluation:** Evaluation of the recognized cell(s) is restricted by MYOCYTER to the cell(s) only, excluding the background. In contrast, MUSCLEMOTION always evaluates the whole frame, regardless of how large the proportion of the moving cell is.

- **Extraction of 43 different parameters:** While MUSCLEMOTION returns only amplitude and speed, MYOCYTER extracts a large variety of parameters including systolic and diastolic share as well as overall contraction time for the applied thresholds (see **Fig. 2**, **panel C**) and the time between two subsequent contractions (**Fig. 2**, **panel B**). From those parameters, detailed statistics for every single cell are also provided. The unique amount of parameters extracted by MYOCYTER can be used to detect statistical differences between different groups (like "untreated control" and "exposed to an agent"), that may be not detectable using MUSCLEMOTION or any other software from this field.

- **Animated output:** This feature provides a video displaying the recognized cell(s) synchronized with the according animated amplitude and is also an unique option only available in MYOCYTER (**Fig. 2**, **panel A**).

- **Dynamic thresholding:** Enables tracking of even very complex amplitudes that are shifted over time and show large differences between the local maxima of single contractions (this may be the result of changed illumination of cells combined with arrhythmic contractions) as shown in **Fig. 4**, **panel C** of the publication.

- **Storage of extensive information:** Results are provided both in graphic and numeric formats that can be used for further statistics; date and time of the evaluation are included as well as the used version of ImageJ and all parameters applied by the user. Thus, the whole process of evaluation can be retracted later, if necessary.

- Using the feature "**Re-evaluation**" (for details, please see the manual in the supplemental material), changed parameters (other thresholds and/or values for "detection") can be applied in real-time to already extracted data without re-evaluation of the original video files, resulting in a very detailed output (both graphic and numeric data).

Table 1: Comparing both features and performance of MYOCYTER and MUSCLEMOTION.

Feature	MYOCYTER	MUSCLEMOTION
Batch processing of multiple files	✓	✓
Numerical extraction of speed and amplitude	✓	✓
Extensive numerical data extraction (including systolic and diastolic share as well as overall peak time for every single applied threshold) of 43 different parameters overall	✓	✘
Detailed both graphical and numerical output of the extracted parameters, also including all four applied thresholds ("Re-evaluation")	✓	✘
Optional video-output of the recognized cell(s) synchronized with the according extracted amplitude	✓	✘
Automatic recognition and evaluation of multiple cells	✓	✘
"Re-evaluation" of already extracted data with changed parameters in real-time	✓	✘
Two different detailed statistics for every evaluated cell (with and without the first and the last contraction)	✓	✘
Automatic detection of the video-framerate	✓	✘
Extracted numerical data is presented clearly in a single file for further processing	✓	✘
"Masked evaluation" for higher precision of the evaluation (ONLY the moving structure evaluated, but NOT the whole frame)	✓	✘
**Evaluation of the included test videos** (i7-4710HQ CPU, 16 GB-RAM, ImageJ 1.52a)	**Evaluation time** [min:sec]	**Evaluation time** [min:sec]
adult cardiomyocyte small.avi	0m:26s (masked and unmasked);	0m:31s
adult cardiomyocyte.avi	1m:04s (masked); 0m:56s (unmasked)	1m:34s
single cardiomyocyte.avi	0m:19s	0m:22s
two cardiomyocytes.avi	1m:16s	N/A, no multi-cell recognition
water flea heart.avi	0m:22s	0m:23s
Faster and more sophisticated evaluation than MUSCLEMOTION	✓	✘

The influence of EtOH on various cardiac contraction parameters of *Daphnia pulex*

Following exposure of *Daphnia pulex* to various concentrations of ethanol (EtOH), various MYOCYTER-quantifiable parameters of cardiac contraction were examined: systole, diastole, total contraction time at two different thresholds, amplitude (averaged from every single local maxima) and the distance between successive maxima (**Fig. Supplement 1, panel A**).

Different concentrations of EtOH show clear effects on the interval between two successive maxima (beat time), which increased by up to 30.3% (at 20‰ EtOH) compared to the untreated control (**Fig. Supplement 1, panel B**). Also at the local maxima of the contraction (averaged over the entire measurement), a drop of 13.4% at the highest EtOH concentration compared to the unexposed control (**Fig. Supplement 1, panel C**) was detected.

Total peak time also increased with increased alcohol concentration (by 29.2% at a threshold of 20% and by 22.3% at Thr50%) as shown in **Fig. Supplement 1**, **panels F** and **I**.

The same was true for systole (up to 36.7% compared to Thr20% control and 30.8% Thr50%), as pictured in **Fig. Supplement 1**, **panels D** and **G**.

The diastole showed a peak value at 5 ‰ ethanol: increase of 28.3% (Thr20%, maximum at 5‰ EtOH) and 25.8% (Thr50%, maximum at 5‰ EtOH) compared to the untreated control (**Fig. Supplement 1**, **panels E** and **H**).

The **panels J** and **K** of **Fig. Supplement 1** represent the ratios of systolic to diastolic times as a function of both different thresholds and changing concentrations of EtOH. While the systolic time increased with also increasing concentrations of EtOH (**Fig. Supplement 1**, **panels D, G**), diastolic time revealed at first a decrease, followed by an increase again (**Fig. Supplement 1**, **panels E, H**), as well as the overall peak time (**Fig. Supplement 1**, **panels F, I**).

These results first show an increase in systolic time at lower alcohol concentrations while diastole shortened. Though, the total peak time increased. At higher alcohol concentrations, there was a greater increase in diastolic time compared to systolic. Thus, by using MYOCYTER it could be shown that not only the contraction time changes, but also their “composition” of both systole and diastole.

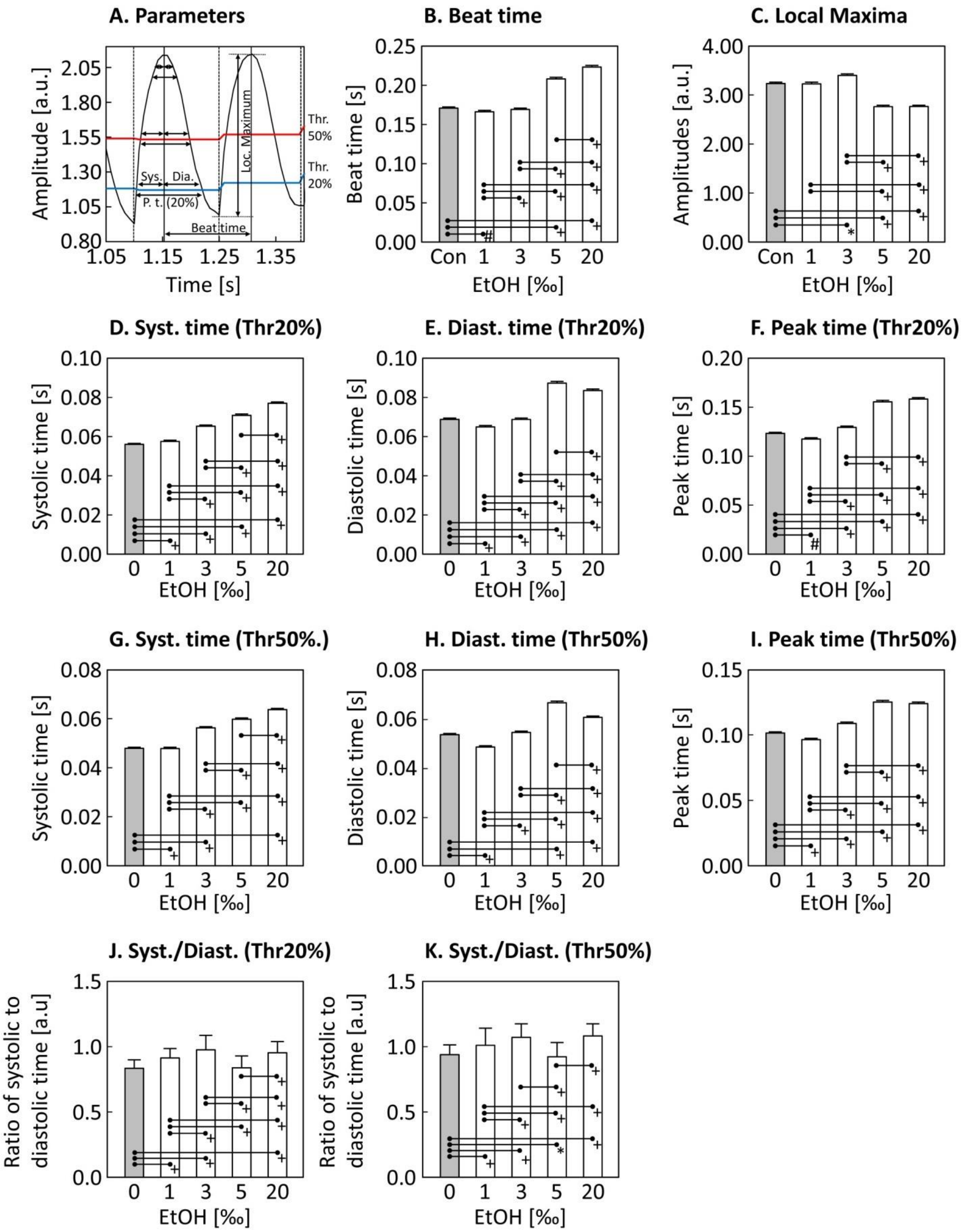
A. Parameters
B. Beat time
C. Local Maxima
D. Syst. time (Thr20%)
E. Diast. time (Thr20%)
F. Peak time (Thr20%)
G. Syst. time (Thr50%.)
H. Diast. time (Thr50%)
I. Peak time (Thr50%)
J. Syst./Diast. (Thr20%)
K. Syst./Diast. (Thr50%)
Amplitude [a.u.]
Time [s]
Thr. 50%
Thr. 20%
Sys.
Dia.
P. t. (20%)
Beat time
Loc. Maximum
Beat time [s]
Amplitudes [a.u.]
Systolic time [s]
Diastolic time [s]
Peak time [s]
Ratio of systolic to diastolic time [a.u]
EtOH [‰]
Con

Fig. Supplement 1: Impact of ethanol on cardiac contraction of *Daphnia pulex*

This figure shows the effects of different ethanol concentrations (EtOH, in ‰) on amplitude, beat time, systolic, diastolic and total peak time in cardiac contractions of water fleas (*Daphnia pulex*).

Panel A displays the measured parameters. The amplitude of two representative contractions (from the control group) is represented as continuous curve. Beat time is the distance between two consecutive maxima (vertical solid line), the amplitude for each contraction is calculated locally as difference between the local minimum (vertical dotted line) and the following maximum. Peak time is the time in which the amplitude is above the corresponding threshold (Thr20%, blue and Thr50%, red). If the amplitude is above a threshold, counting of the according systolic time begins, after exceeding the local maximum the diastolic time starts, while the sum of both returns the total peak time. **Panel B** shows the effects of different EtOH concentrations (1, 3, 5, and 20 ‰ in comparison to an unexposed control, “0”) on the beat time (distance between two consecutive maxima) of the heartbeat of water fleas. **Panel C** shows the corresponding amplitudes. The **panels D-F** display the systolic, diastolic and total peak times of a contraction for a threshold of 20%, the **panels G-I** display the corresponding values for a threshold of 50%. The **panels J** and **K** represent the ratios of systoles to diastoles depending on alcohol concentration for the indicated thresholds (20% and 50%).

Statistics: significant differences are indicated by connecting lines; *: ($p<0.05$), **: ($p<0.01$), #: ($p<0.005$), and +: ($p<0.001$); one-way ANOVA.
