# Supplementary material for: The “MYOCYTER” – Convert cellular and cardiac contractions into numbers with ImageJ
Source: Sci Rep. 2019 Oct 22;9:15112. doi: 10.1038/s41598-019-51676-x (PMC6805901; doi:10.1038/s41598-019-51676-x)
Supplement: Supplementary file 2 — Manual for MYOCYTER [file 41598_2019_51676_MOESM2_ESM.pdf]

Online Supplement

The “MYOCYTER” – Convert cellular and cardiac contractions into numbers with ImageJ

MYOCYTER – An analysis tool to convert cellular and muscle tissue contractions into numerical and graphical data with ImageJ

Tilman Grune[1,2,3,4,5] MD, Christiane Ott[1,3] PhD, Steffen Häseli[1] B.Sc, Annika Höhn[1,2] PhD, and Tobias Jung[1,3] PhD

[1] Department of Molecular Toxicology, German Institute of Human Nutrition Potsdam-Rehbruecke (DIfE), 14558 Nuthetal, Germany
[2] German Center for Diabetes Research (DZD), 85764 Muenchen-Neuherberg, Germany
[3] German Center for Cardiovascular Research (DZHK), 10117 Berlin, Germany
[4] NutriAct – Competence Cluster Nutrition Research Berlin-Potsdam, 14558 Nuthetal, Germany
[5] University of Potsdam, Institute of Nutrition, 14588 Nuthetal, Germany

Correspondence: Tobias Jung, Department of Molecular Toxicology, German Institute of Human Nutrition Potsdam-Rehbruecke (DIfE), Arthur-Scheunert-Allee 114-116, 14558 Nuthetal, Germany; e-mail: tobias.jung@dife.de; phone: +49 (0)33200 88-2490

MANUAL

MYOCYTER v.1.0 – User Manual

Tobias Jung

Correspondence: Tobias Jung, Department of Molecular Toxicology, *German Institute of Human Nutrition Potsdam-Rehbruecke* (*DIfE*), Arthur-Scheunert-Allee 114-116, 14558 Nuthetal, Germany; e-mail: tobias.jung@dife.de; phone: +49 (0)33200 88-2490

July 2019

Table of Contents

What is "MYOCYTER"? 2

Quick start guide 2

How does MYOCYTER work? 4

What is necessary to run MYOCYTER? 5

How to get video files from a microscope? 6

How to start MYOCYTER in ImageJ? 8

Preparing your video files for MYOCYTER 10

Video compression 11

Processing your videos in MYOCYTER – Pretest 12

Processing your videos in MYOCYTER – Evaluation 16

The numeric output of MYOCYTER 20

The graphical output of MYOCYTER 26

Further processing of the numerical output 27

  How to import the data 28

  How to adjust the settings 33

Interpreting the data 35

Adapt the code 35

Additional material 36

A complete representative evaluation 37

What is “MYOCYTER”?

MYOCYTER is a macro for ImageJ (**for v1.52a and above**), which extracts a wide range of information from video files of cyclic contracting cells such as cardiomyocytes or even whole hearts.

The macro outputs a spreadsheet with 42 different parameters, which are measured over time for each single frame of the video, as well as detailed statistics on the data obtained. The results are stored as tab-delimited text files that are compatible with traditional software for further data processing, analysis and presentation such as “Excel”, “SPSS”, “SigmaPlot”, or “Prism”.

The code of MYOCYTER is open-source (written in the most used Java-like programming language), contains detailed descriptions of the individual subsections, which greatly simplify individual modification or adaptation of the macro(s) by the user, if necessary. The data output contains a detailed data table, images and (optionally) animations.

Quick start guide

- Provide your movies in a separate folder (*.avi-format, either uncompressed or MJPEG-compression, the extension has to be “.avi”, also in lower case)
- Start ImageJ (v. 1.52a or higher)
- Load MYOCYTER by dragging and dropping the txt-file containing the macro on the ImageJ-window
- Start MYOCYTER (Macros>Run Macro) or (Strg+R)

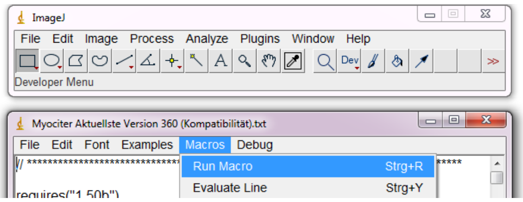

- Pretest your videos:

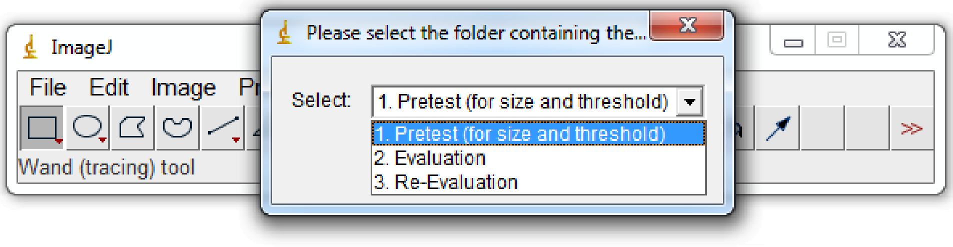

- Start with “LowMovement”

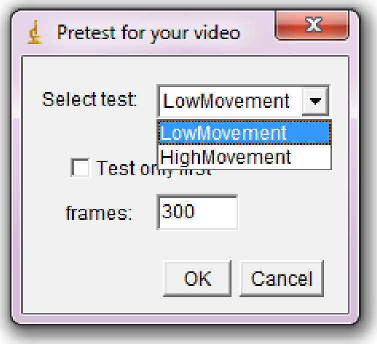

- After the pretest you should have something like this:

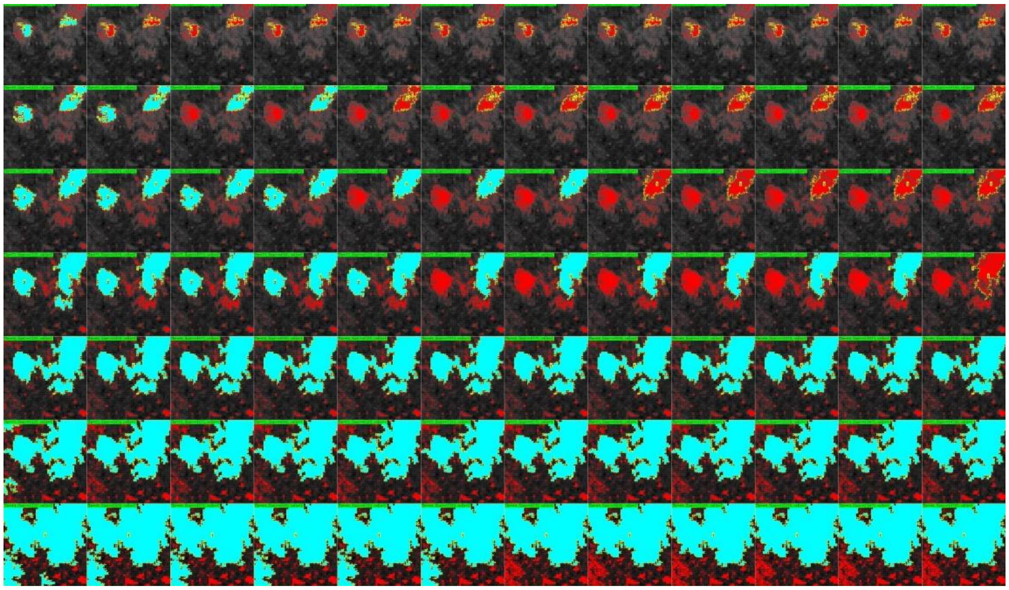

- Take the settings for threshold and size (found in the small images) that match you cells as good as possible and apply them during evaluation of the videos. The option “video output” may not be necessary and can be deactivated, leave the other parameters as they are (for now). Klick “OK” to start evaluation.

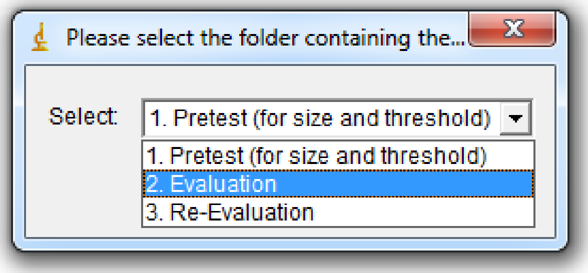

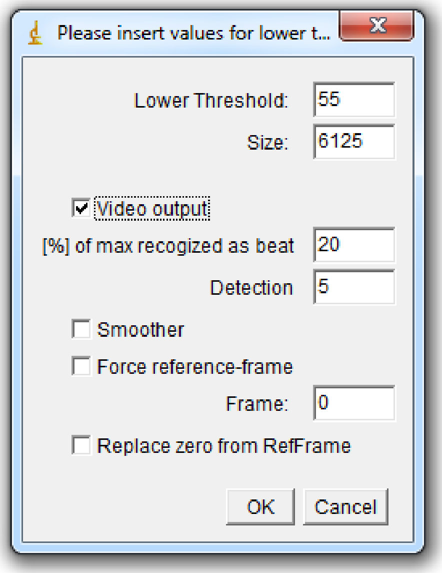

In the following, the exact function of this macro as well as of every single parameter is discussed in detail.

How does MYOCYTER work?

The basic idea of the macro is very simple:

If one has a video of contracting cardiomyocytes (or similar moving structures of interest), their movement induces differences between the successive images of the corresponding video. If the cell is static, the differences are very small, if the cell moves quickly, the differences are correspondingly higher. These differences between the individual images can be easily measured with a function implemented in ImageJ, the result is presented as intensity map: Brightness represents big differences (=fast movement), and darker areas indicate little or no differences (=less/no movement). Pixel intensity correlates with the movement of the cells and can easily be measured with ImageJ.

Two types of differences are measured: one between the successive frames of the video, the other between a reference image ("reference frame", more on this later) and all other images in their chronological order.

The differences between successive images correspond to the "speed" of a movement: the speed will be high, if a cell moves quickly and the resulting differences between successive frames will be also high.

Differences between a reference image and all other images in their temporal order correspond to the "amplitude" of the movement, as the "amount of deformation" of the contracting cell compared to the reference image. If the reference image represents the cell in its resting state, the amplitude will be low if the reference frame is compared to other frames showing the cell in its resting state. But if the reference image is compared to the cell in its state of maximum contraction, the difference will be high and consequently the amplitude also.

If one wants to represent the amplitude such that a high value also corresponds to a high contraction, then the reference image should represent the cell in the non-contracted resting state.
The reference image can be selected in two different ways: Either the macro automatically calculates it by determining the image with the least difference to the next one. Empirically, it has been shown that these images are usually in the resting phase of cells or hearts. Or the user selects the corresponding picture by entering the according frame number (see later).

If one selects a picture of the cell in the state of maximal contraction as reference picture, the classical graph becomes inverted: states of maximal contraction are now represented by the minima of the curve, which represents the differences to the reference picture. The basics of such motion analysis are described in detail by Jan David Kijlstra (*Stem Cell Reports*, **5:1226-1238**, 2015). Custom or automatic selection of the reference image will be discussed later in the section "**Processing your videos in MYOCYTER – Evaluation**".

After extracting the values for "amplitude" and "speed" over time from a video file, a large variety of further parameters is also calculated from these date by setting various thresholds and via detection of local maxima and minima.

What is necessary to run MYOCYTER?

- You need ImageJ (version 1.52a or higher, freeware)
- You need video files that are compatible with ImageJ (uncompressed or MJPEG-compression)
- You need "MYOCYTER".

ImageJ

To execute this macro, the user needs ImageJ, a public domain, Java-based image processing software, available here (for free):

https://imagej.nih.gov/ij/download.html

Though, ImageJ and FIJI are considered to be able to run the same macros, but in this case, the **use of FIJI is NOT recommended**. MYOCYTER is **tested and optimized for ImageJ 1.52b and use of this (current) version is highly recommended**. FIJI uses a different naming of auto-generated image windows that is not (always) compatible with ImageJ. If MYOCYTER is executed in FIJI, it is likely that the macro will abort with the message "image not found in line xy".

However, since ImageJ is freeware, it is no problem to get the corresponding version 1.52b (or higher).

MYOCYTER

This macro and additional material (sample videos) can be downloaded at www.scyrus.de, just visit the macro section. Any updates or modifications of MYOCYTER released by our group will be also found there.

How to get video files from a microscope?

The minimal experimental set-up consists of a transmission light microscope, a holder (there are many different types available) attaching a current smartphone to the ocular and the smartphone itself. The smartphone should have a “slomo” function and be able to produce videos with more than 100 frames per second (> 100 fps) - but this is the case for most of the current mid-range smartphones (120-240 fps). 100 frames per second should be considered as the recommended lowest limit for a meaningful evaluation. Cardiomyocytes in cell culture contract about 2 times per second. Thus, a recorded contraction is only represented by about 50 images (rather less). However, these 50 measurement points are necessary to map the contraction in detail and to examine, for example, changes in systole and diastole. Mouse hearts beat about 10-12 times per second – so, there are less than 10 measuring points or single images depicting a whole contraction. In order to obtain 40-50 measuring points in this case, too, a correspondingly higher frame rate of around 500 fps would be necessary. In shrews, the heart rate can be up to 22 beats per second, which in turn would increase the required frame rate to nearly 1000 frames per second. The hardware used in this case should be adapted to the examination object if necessary. A well known manufacturer announced in 2017 the coming release of smartphones that should allow frame rates of almost 1000 fps.

The effects of a low frame rate on the depiction of a contraction are shown in **Fig. 1**.

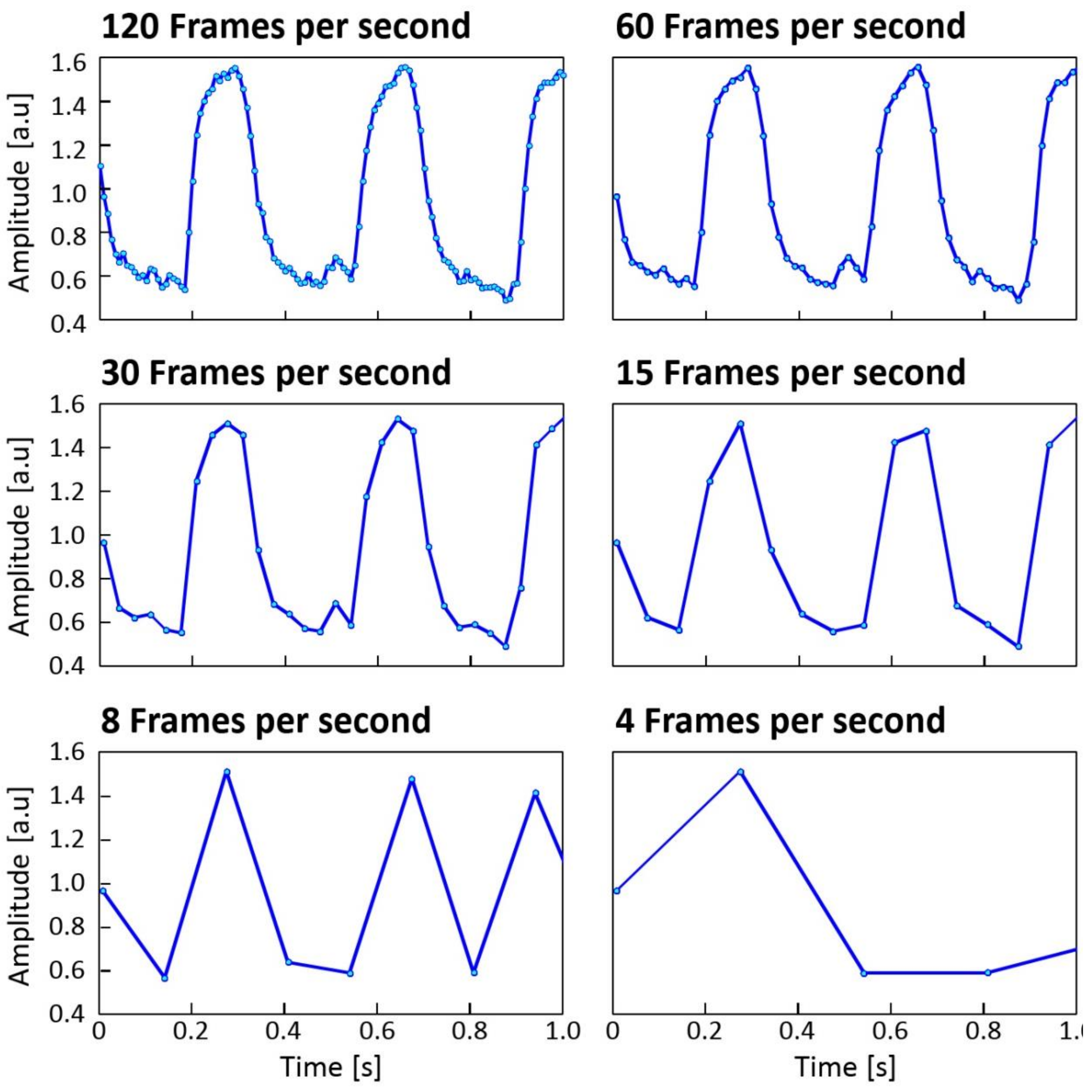

Fig. 1: Effects of the frame rate on the calculated amplitude

This figure shows the effects of the frame rate on the final plot of the amplitude after evaluation with MYOCYTER. A cardiomyocyte (isolated from a mouse) contracts about 3 times per second, so at 120 frames per second there are about 40 data points/frames per contraction available ("120 fps"). If the frame rate of the picture is reduced (halving from panel to panel), correspondingly fewer measuring points are available for describing the shape of a contraction. The shape of the curve loses detail until a complete contraction is only represented by a single measurement point (at "8 fps"). At "4 fps", the frame rate is so low that the information of whole contractions is missing.

Please also keep in mind when choosing the hardware that you may spend a whole day at the microscope. If your smartphone is charged via the USB port of a computer, then its output voltage/output power may not be sufficient to charge the smartphone faster than it is unloaded by video recording. USB 2.0 provides 500 mA at 5 volts, USB 3.1 (900mA/5V) and USB Type-C (1.5 to 3A at 5V). With an older USB port, it may be necessary to provide a portable charger,

which charges the smartphone faster, so that the video recording does not have to be interrupted for a longer time.

It is also possible that during an experiment, several hours of high-resolution material (HD, Full HD, 4K) at very high frame rates (100-1000 fps) incurred and the corresponding amount of data may be larger than 100 gigabytes. In this case, the data from the smartphone should already be moved to a computer or mobile data storage during the experiment.

How to start MYOCYTER in ImageJ?

The simplest way:

1. Start ImageJ (**Fig. 2**)
2. Drag & drop the text file containing the macro directly onto ImageJ (**Fig. 3**)
3. Select “Macros” > “Run Macro” in the new window or just “String + R” on the keyboard.

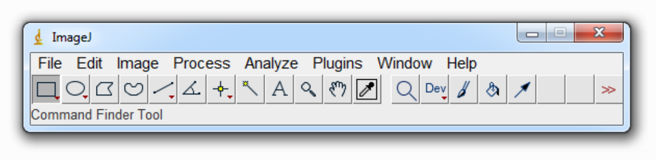

Fig. 2: The user interface of ImageJ

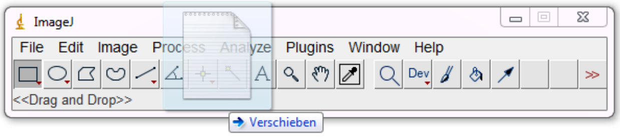

Fig. 3: Drag and Drop the text file containing the macro “MYOCYTER”

If this does not work (for any reason), open the text file containing the macro, select all (*String + A*) copy the macro (*String + C*). Open ImageJ, select “Plugins” > “Macros” > “Record”, paste the macro in the new window (*String + V*) named “Recorder”, click “Create” (**Fig. 4**) and in the new window select “Macros” > “Run Macro” (**Fig. 5**) in the new window or just press *String + R* on the keyboard.

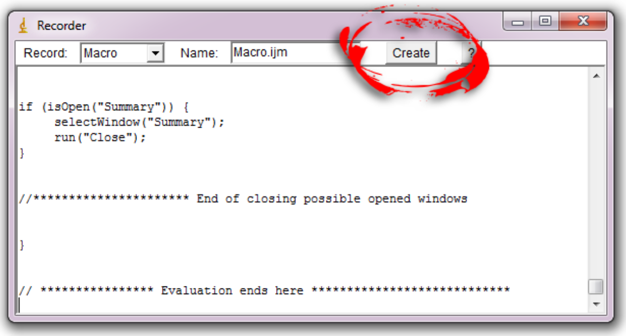

Fig. 4: **Recording and creating a macro**

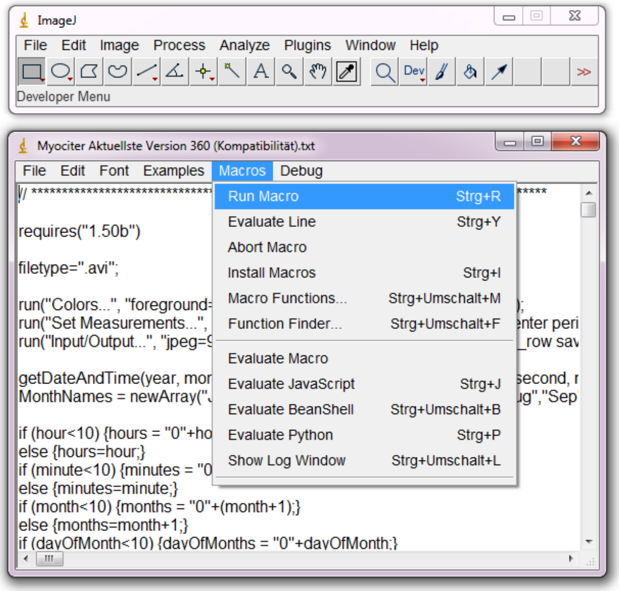

Fig. 5: **Starting a macro in ImageJ**

After starting the macro, the user can select between "**1. Pretest**", "**2. Evaluation**", and "**3. Re-Evaluation**".

"**Pretest**" processes all videos in the folder that is selected and returns a tiled image with different combination of the size of moving structures ("**Size**") that the user want to be recognized as cells (increasing horizontally) and intensity "**Threshold**" (corresponding to the intensity of cellular moving, changed vertically). The number of cells (ROIs) recognized in the original video does not matter here - it can be a single one or several cells, since every single ROI is evaluated separately.

If one combination of “**Size**” and “**Threshold**” matches the needs of the user, the according video is processed by selecting “**2. Evaluation**” followed by specification of the appropriate settings after restart of the macro.

Evaluation returns a large variety of information extracted from the video file: The first one is the first frame of the analyzed video with information about the investigated ROIs, outlined in yellow and marked with numbers. While saving, the individual data of the according ROIs are identified with the filename extension “cell 01”, “cell 02” and can be easily assigned to the image displaying the ROIs. Also, every single ROI is stored as separate video file extracted from the original one, as well as video just displaying the difference between subsequent frames. In a separate subfolder, a plot showing the amplitude of the according ROI is stored (in two different resolutions).

Besides of the static images, also an animation is generated (this is optional), that shows the complete plot scrolling from left to right with the moving cell from the according ROI in the upper left corner at exactly the moment, where the scrolling plot touches the y-axis, while the x-axis represents the according time in seconds (the first frame is defined to be at time point zero), calculated from the frame rate of the original video. This animation can be opened later in ImageJ for frame by frame view. Finally the data are also stored as data table (text file, tab delimited) containing the filename of the analyzed video, the specific ROI, and all the data columns extracted from the video file(s). At the end of this table, several statistics are shown. Furthermore, every evaluation is stored with date, time and the settings applied.

Preparing your video files for MYOCYTER

After recording with a smartphone or video camera, most of the files will be in MOV or MP4 format. ImageJ is (yet) unable read these formats. Thus, you need a video converter that converts this file type into an ImageJ-readable format. Readable for ImageJ are uncompressed AVI files, which, however, occupy a great amount of RAM during data processing. Another ImageJ-compatible format are MJPEG-compressed AVI files.

But even if material is sufficiently high frame rate, there may be other technical problems that affect the evaluation. MOV and MP4 are already lossy compression formats, so converting them to another lossy format (MJPEG) can significantly (further) reduce video quality. Another source of artifacts are so-called keyframes. One strategy of (lossy) video compression is storage of a whole frame (keyframe) followed by storage of just the differences between subsequent frames for the next 100-200 frames before resetting a whole keyframe. The difference between a

normal image and a keyframe can be significantly larger than between the images preceding and following that keyframe. Such a deviation can be misinterpreted as movement during evaluation via MYOCYTER.

IMPORTANT:

- Avoid the special character “-“ in the filenames of the videos to be analyzed.
- The extension of the file should be “.avi”, but NOT “.AVI” (or else). This issue can be solved quickly by using the Windows command line (cmd.exe). Select the according folder, type “ren *.AVI *.avi” (without the quotation marks) and press “ENTER”.

Video compression

ImageJ is very dependent on memory. For very large files, it may be advantageous, depending on the existing hardware, to shrink or shorten these files. Furthermore, it has been shown that compression of video data can have a significant influence on the results (**Fig. 3**, **panels I** and **J**), especially if the area of the cell to be examined is small compared to the overall picture. Since most cameras and smartphones record videos in MP4 or MOV format, which are lossy compressions (in contrast to lossless compression, groups of similar pixels are merged, and information is actually lost), these effects are already present in the input data. The recording of uncompressed video data in the resolution that today’s mid-range smartphones already offer (HD, Full HD) at frame rates of 120 or 240 fps would require very fast hardware and produce extremely large amounts of data (uncompressed AVI, 120 fps, HD, >332 MB/s, in contrast, MOV from a smartphone, 120 fps, HD, is recorded at about 900-1000 kB/s in a typical video). Thus, at least for the moment, further compression (MJPEG) of already compressed data (MP4/MOV) is necessary to provide videos that are compatible with ImageJ. This should be taken into account if videos from different sources or after different processing are evaluated and compared.

Another source of artifacts are so-called keyframes. One strategy of video compression is to store a whole frame (keyframe) and then just frame-to-frame changes for the next 100-200 frames before resetting a keyframe. The difference between a normal image and a keyframe may be significantly larger than between the images preceding and following that keyframe. Such a deviation can be misinterpreted as movement during evaluation. Though, this issue is an integral aspect using current smartphones, according to our experience (data not shown), the mentioned effects are negligible small compared to the differences of subsequent frames that are induced by (cellular) contractions.

Processing your videos in MYOCYTER – Pretest

IMPORTANT:
Please do not use the computer otherwise while MYOCYTER is running. ImageJ puts the current window always in the foreground and simultaneous user input into other programs is therefore hardly possible. Macros that run with ImageJ can be accidentally canceled while using other programs at the same time. Please wait until MYOCYTER has finished the evaluation and all open windows have been closed automatically.

After starting the MYOCYTER, you will be asked to select:

1. Pretest (for size and threshold).
2. Evaluation
3. Re-Evaluation

Let’s start with “**1. Pretest**” (select it and click “Okay”) as shown in **Fig. 6**. The pretest is only necessary if the video contains several cells or structures, which should be examined independently. If this is not the case, if the video contains only a single “region of interest”, the pretest can be skipped. Though, the pretest may significantly enhance the precision of recognition.

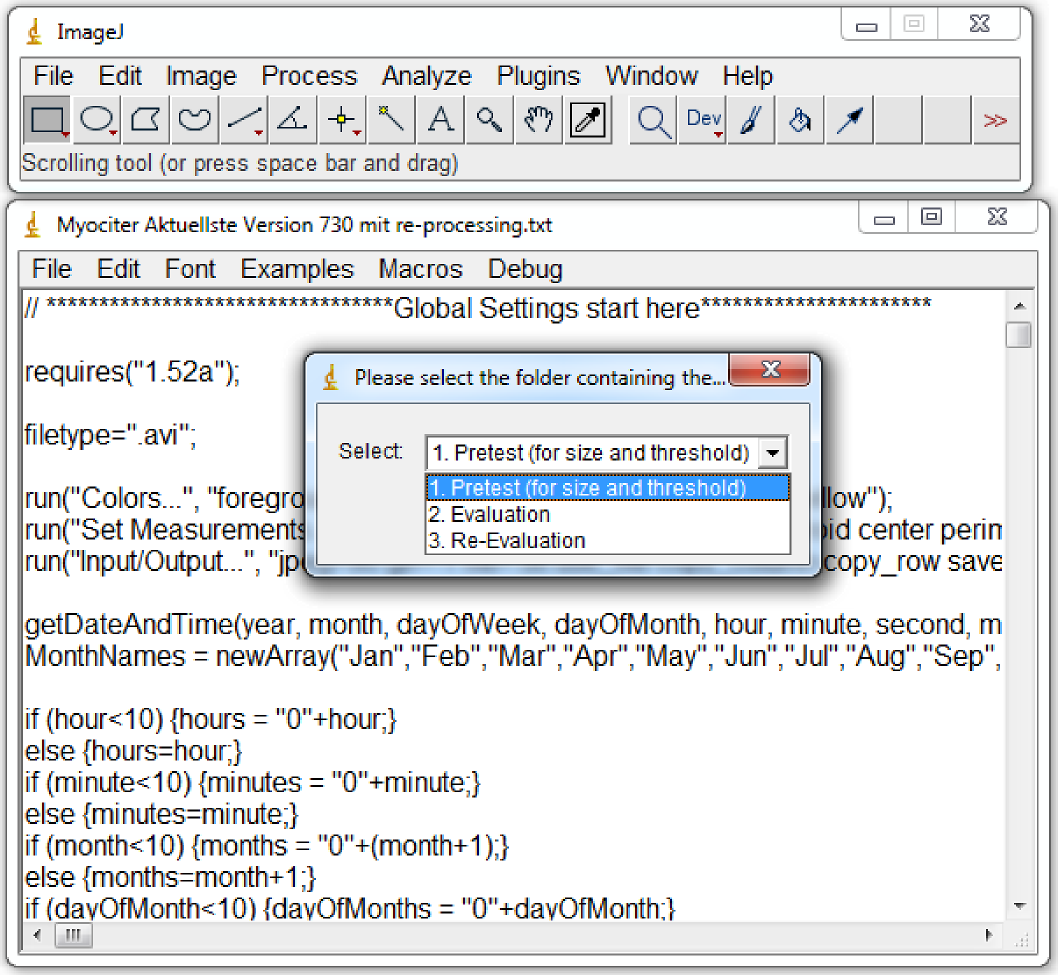

Fig. 6: After starting MYOCYTER, the user is asked to select either “Pretest” or “Evaluation”. First, we start with “Pretest”.

Now, you will be asked to select between “**LowMovement**” and “**HighMovement**”. For the beginning, keep at “**LowMovement**” (**Fig. 7**) for cells moving at an “average”.

These settings optimize the pretest for the “average” movement expected of cardiomyocytes in light microscope.

Then select the folder (just select, **do not open** it) your videos are stored in and click “OK”. The pretest begins and is executed for every single video. If the videos are very long, or very large (ImageJ is quite dependent on the RAM of the computer used), or if there are many videos for pretesting, it is possible to limit the pretest to any number of frames from the beginning of the video. However, this number should not be too low for good recognition (**Fig. 7**) and ≥30% of the whole frame number may be a good start.

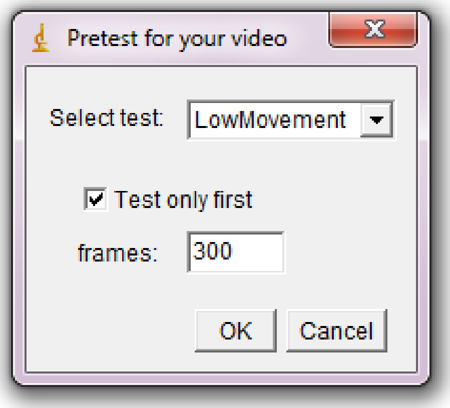

Fig. 7: Selection of "Low Movement".

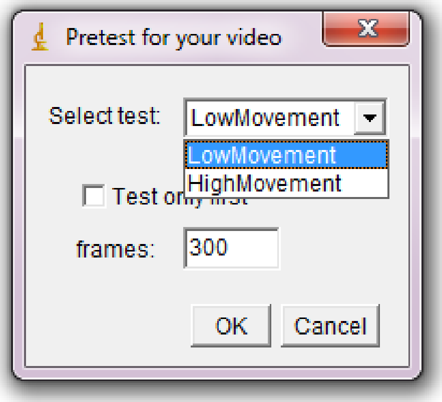

Fig. 8: In the case of many or very large files, it may save time testing only a few hundred frames from the beginning of the video.

After completion of the pretest, the corresponding results (images) are stored for every single tested video file in a newly created subfolder. Such a (typical) pretest image is shown in **Fig. 9**.

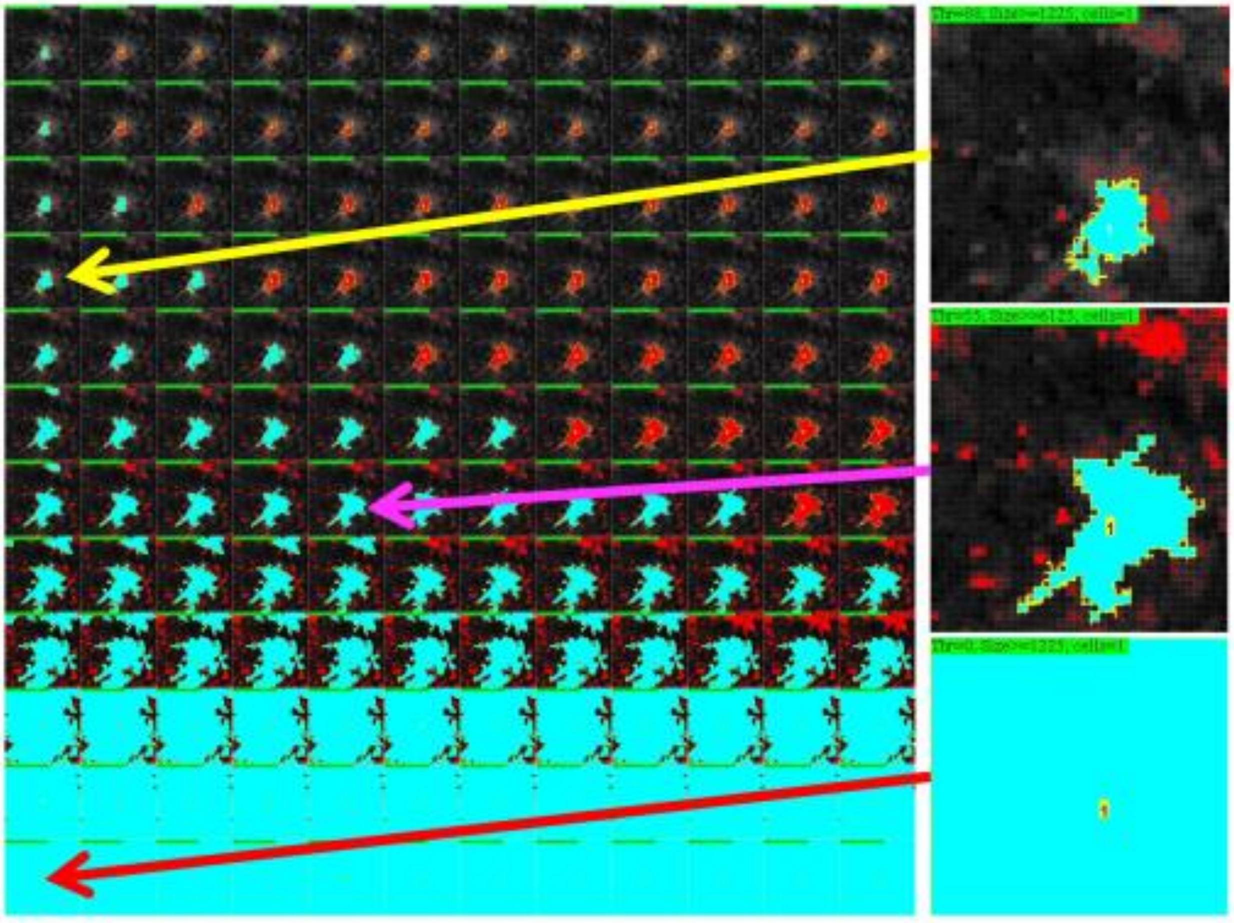

Fig. 9: MYOCYTERs summary of the "Pretest" (left part of the figure).

Different combinations of particle size ("Size") and threshold ("Thr") are applied to the sample. "Cells" (in this case only a single one) just returns the number of cells that are recognized using the according settings. The settings best matching/covering the moving cell (represented by the yellow-outlined light blue area) are applied for final evaluation. The yellow arrow points to the structure that is recognized using a threshold of 88 and a particle size of 1225 (the minimal area of particles that are recognized in pixels). This narrows the "region of interest" to the part of the cell(s) with the highest movement. The purple arrow points to a threshold of 55 and a particle size of 6125. Using this settings, the whole cell is recognized and evaluated. Parts that are not marked in light blue are not included in the evaluation. The red arrow points to a combination of parameters using a threshold of 0. In this case, the whole frame is evaluated, all parts of the image, moving or not, are quantified.

Please choose the two values for "Lower Threshold" and "Size" from **Fig. 9** that best fit your sample. Then start MYOCYTER again and select the option "Evaluation" (**Fig. 10**).

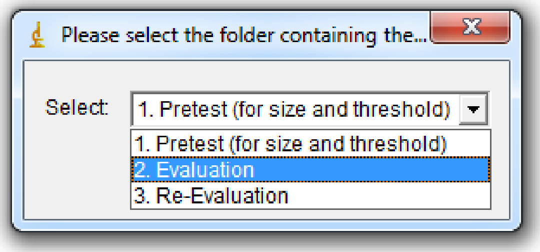

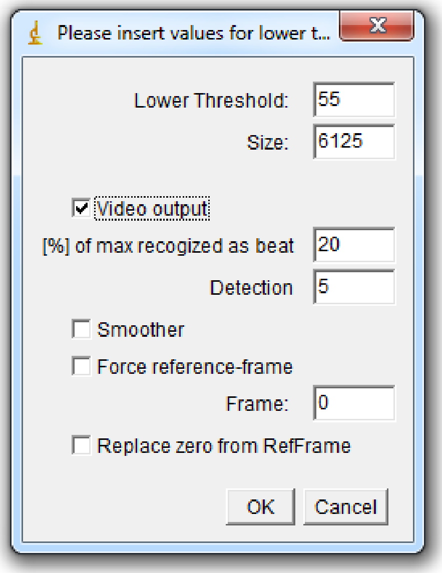

Fig. 10: Select "Evaluation" to evaluate your data.

Fig. 11: Settings for the evaluation of a video file.

Processing your videos in MYOCYTER – Evaluation

In this case, according to the results of the pretest that are depicted in **Fig. 9**, we select the values 55 for "**Lower Threshold**" and 6125 for minimum cell "**Size**" to be recognized (as shown in **Fig. 11**).

In the following, we take a look at the available settings for evaluation of a video file.

The option "**Video output**" remains deselected in most cases; when it is selected, among other things, a video is produced which represents the amplitude of the selected cell synchronized with the corresponding cell itself. This feature is primarily intended to control the evaluation as well as to present selected data. For the evaluation of very many cells, this option is not recommended, especially since it massively increases evaluation time. Nevertheless, the feature "**Video output**"can be very useful and descriptive.

"**[%] of max recognized as beat**" is set at 20% (default). This is the user threshold and in this case means that an amplitude exceeding 20% of the following maximum (to be precise: exceeding the current detected minimum + 20% of the following maximum) is recognized as a peak. In addition to the user threshold, three additional thresholds are automatically applied to the data: 10%, 50%, and 90%.

"**Detection**" (**Fig. 11**) indicates the sensitivity used for detection of maxima and minima. For most data, where the difference between baseline (the cell is not contracted) and maximum (the cell is maximally contracted) is high, values between 5 and 10 are perfectly adequate. In this case, the maxima (the local maxima of the amplitude) of the individual contractions are very similar. In special cases, such as arrhythmia, in which the differences between the individual maxima can be very strong, it is better to choose a higher value. This can be in the range of 20-30 and thus even smaller differences in the amplitude are recognized as local maxima and minima. If the value for "Detection" is set too high, eventually even noise is recognized as a sequence of separate contractions. After some evaluations, this is an empirical value that can be well estimated by the user. If the value is still wrong, you can analyze the extracted data in real time in a further step without having to reanalyze the whole video (more on that later in the chapter "*Further processing of the numerical output*").

"Smoother" (**Fig. 11**) is a function that enables automatic detection and elimination of individual maxima which deviate extremely from all other detected maxima and result, for example, from a vibration of the microscope during the measurement. In most cases this option is not necessary.

"Force reference frame" (**Fig. 11**) allows the user to select a particular frame of the video as the reference image. If this option is not selected (default setting), MYOCYTER automatically determines the reference image of the video: the image which has the smallest difference to the following image is selected as the reference image. Experience shows that this picture is (mostly) in the resting phase between two contractions. The amplitude thus appears as a positive peak, while the resting state of the cell represents the baseline. Usually, MYOCYTER automatically recognizes the appropriate reference image in the resting state of the cell. However, if this is not the case, for example in case of severe arrhythmia or cardiac contractions without any visible resting phase, then it is also possible to force a reference image. The impact of the reference frame on the amplitude generated from the data is depicted in **Fig. 12**.

If more than one cell is recognized, the reference frame is determined for both cells independently. Though, if the reference frame is forced, the same frame would be applied to both cells – thus, it is easy to see that forcing the reference frame requires only a single structure in the analyzed video.

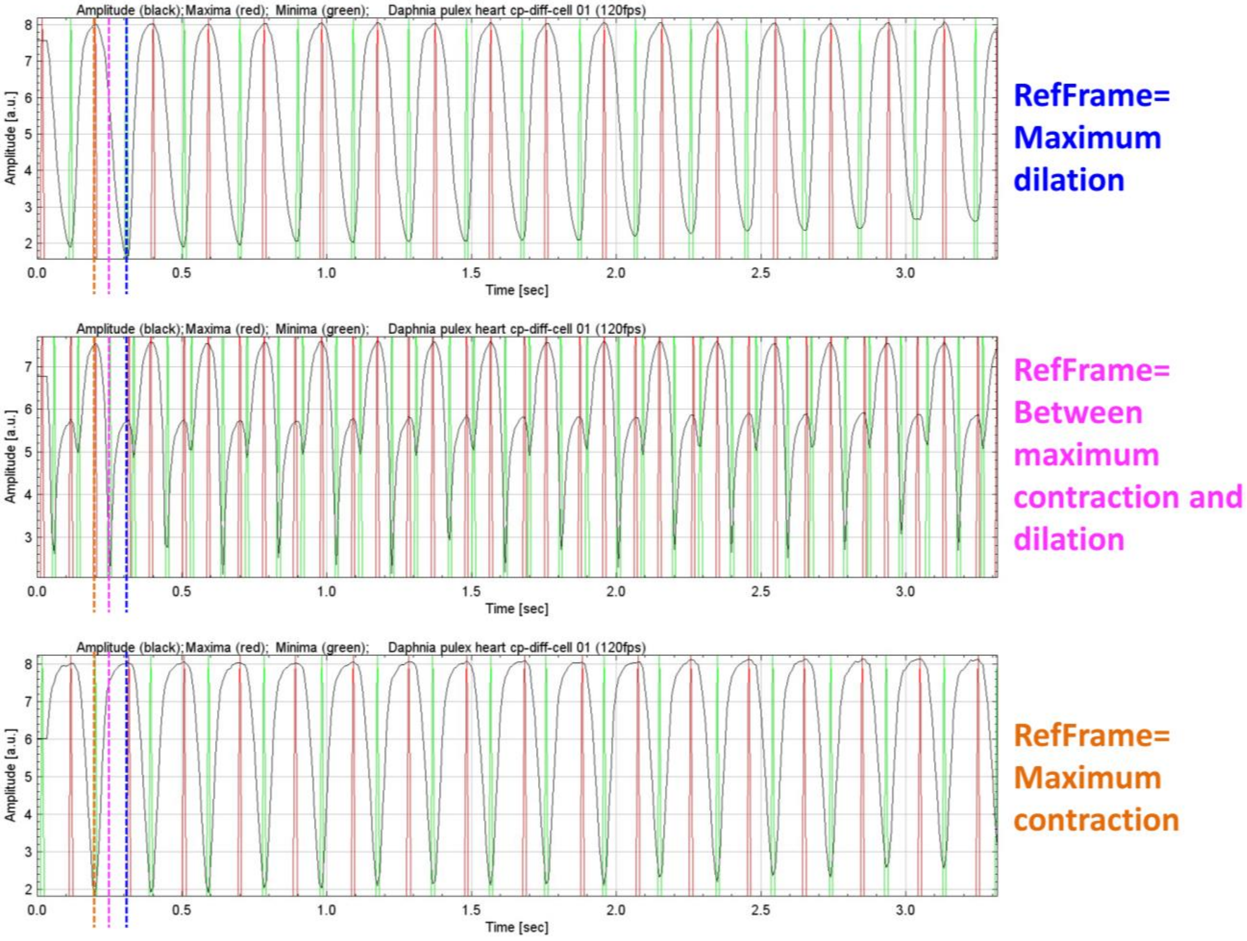

Fig. 12: Selection of the reference image

The selection of the reference image determines the shape of the final amplitude. If the state of maximum dilatation of the cell/object is selected (blue, upper partial image) the contraction of the heart of a water flea (*Daphnia pulex*) shows the according amplitude form. During a cyclic movement, the heart will always return to the state of maximum dilatation and the corresponding images of the film have only a small deviation from the reference image: the amplitude indicates a minimum whenever the cell most closely resembles the state of maximum dilatation. If the user selects the state of maximum contraction (lowest partial image, orange) then the minimum of the amplitude always appears where the cell most closely resembles the state of maximum contraction. Depending on which of the two states are chosen as a reference image, maxima and minima on the top and bottom panels are reversed.

If one chooses as a reference image a state between maximum contraction and maximum dilatation, the result is an amplitude curve as shown on the middle panel, and the amplitude accordingly contains a “double peak”. The minima of the amplitude in this case represent the state of the cell between maximum and minimum contraction. When the reference image is automatically selected, MYOCYTER usually selects a picture from the resting phase of the cell/object (at the state of maximum dilatation).

“Replace zero from RefFrame” (**Fig. 11**) replaces the zero value of the amplitude at the position of the reference frame (the difference between the reference image and itself is of course zero) by the next lower global value of the amplitude of the evaluated object. The effect of this checkbox is depicted in **Fig. 13**. The lowest global difference between two subsequent frames is likely to be in the order of the noise of the video file. Thus, it seems to be legitimate to replace a zero with a value in the order of noise.

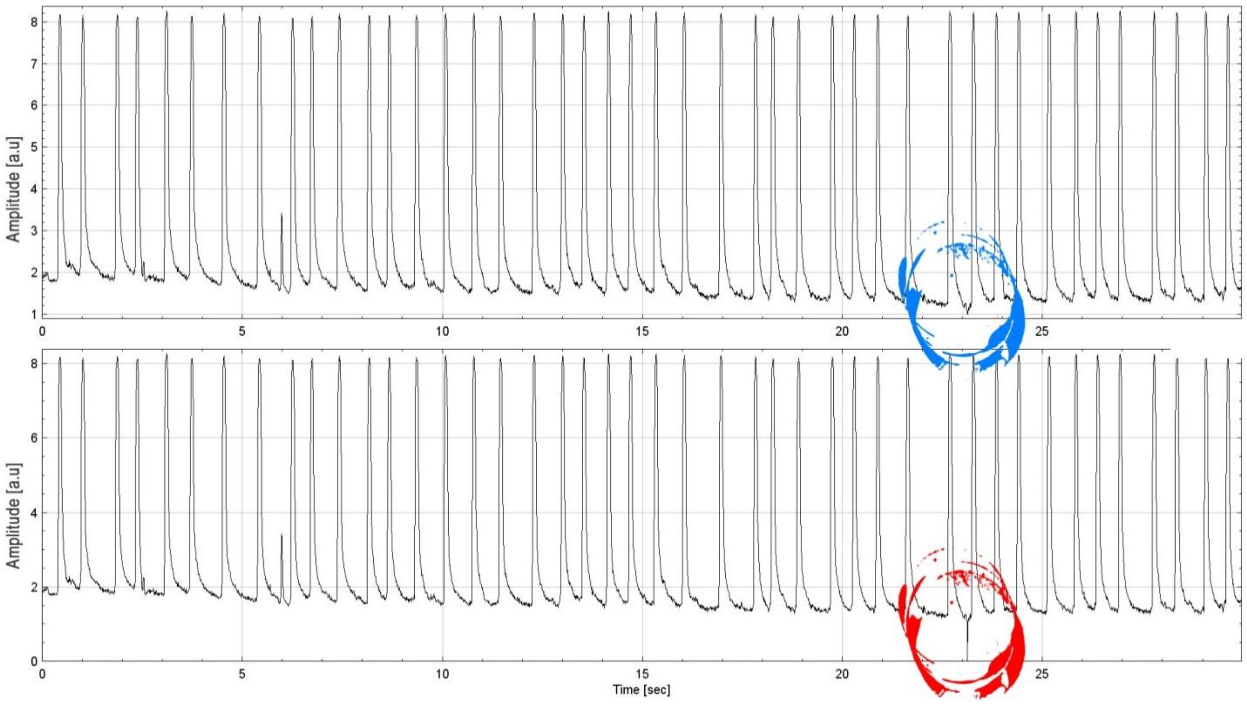

Fig. 13: Effects of replacing the zero of the reference frame.

The amplitude represents the difference between each frame of the entire video and the reference frame. Of course, since the difference between the reference frame and itself is zero, the corresponding amplitude at this point also indicates a zero value (lower part of this figure, red circle). If one replaces this “RefFrame-zero” by the smallest global value of the amplitude, the plot changes according to the upper partial image (blue circle).

Now, the evaluation of the video is started by clicking on **“OK”** (**Fig. 11**) and may take several minutes.

The numeric output of MYOCYTER

MYOCYTER creates a subfolder in the video folder that contains the results and in its name both date and time of the corresponding evaluation. Again, this folder contains several subfolders. First, there is an overview image, which shows the recognized cells numbered. Such a sample image is shown in **Fig. 14**.

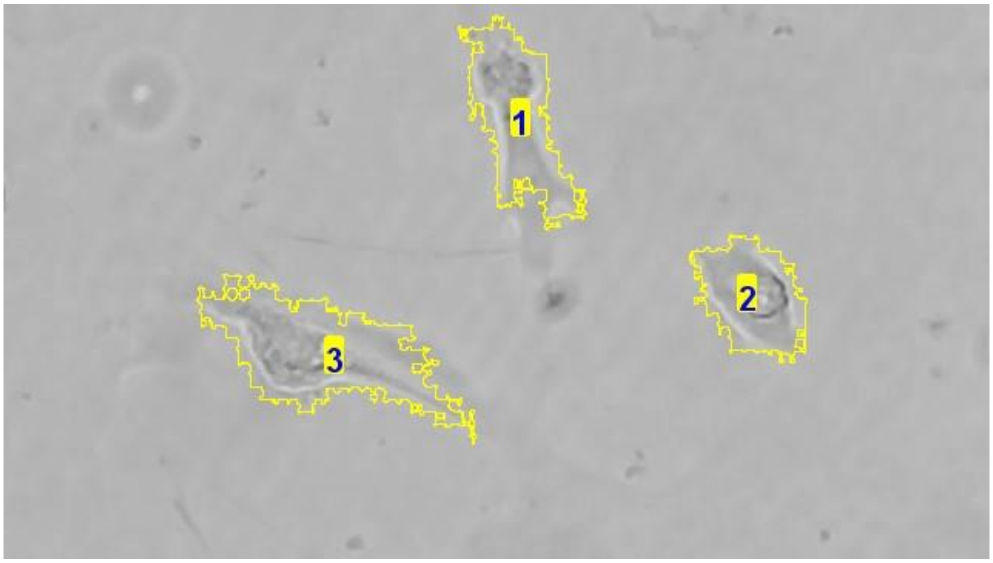

Fig. 14: Overview of the detected cells or moving structures.

MYOCYTER produces an overview image for each analyzed video showing all detected cells or moving structures (outlined in yellow and numbered). The numbering is important in order to assign the results later, the naming is done in the format "filename-cell number". The angular shape of the yellow border is a compression artifact. The problem of compression will be discussed later in section "*Preparing your video files for MYOCYTER*".

The first subfolder is called "isolated" and contains the individual cells recognized in the original video, each as a sectioned video limited to the according regions. The second subfolder is called "diffMov" - and contains the difference videos of the detected single objects. These difference videos represent the differences between successive images. Pixels that differ greatly are displayed brighter, while pixels that differ slightly are darker. Again, there is a subfolder called "dataplots". This subfolder contains among other things the numerical output of MYOCYTER.

The text file "Results" with date and time of the evaluation in its name contains the entire numerical output as a tab-delimited table. This table should be compatible with most popular statistics programs (such as Excel, SPSS, Prism, SigmaPlot). At the beginning of each data table

the file name of the corresponding video and the region/cell which is displayed (see overview picture in the main directory). Following is a table listing 42 different parameters for each video frame.

Here, the measured parameters are listed:

- **Frame** – Absolute frame number (integer) of the analyzed video.
- **Time [sec]** – Absolute time in seconds computed according to the frame rate of the analyzed video (ImageJ extracts this parameter from the file).
- **Amplitude [a.u.]** – Amount of difference between the current frame and the defined or computed reference frame (**Fig. 15**).
- **Amplitude baselined [a.u.]** – Amplitude after subtraction of the lowest global amplitude value.
- **First derivation of amplitude [a.u.]** – Difference between the reference frame and the current frame.
- **Absolute of the first derivation of amplitude [a.u.]** – Just the absolute values of this parameter.
- **Speed [a.u.]** – Difference between frame (n) and (n+1) at every single time point (this output is always ≥0).
- **Speed baselined [a.u.]** – Speed after subtraction of the lowest global speed value.
- **Maximum/Minimum [a.u.]** – Returns only a number at the exact location of the maximum/minimum, while being zero at every other point. The number returned is the global maximum of the amplitude rounded to the next integer, thus fitting the dimension of the plotted data.
- **Maxamplitudes [a.u.]** – The local amplitude is calculated as the difference between the current minimum and the next maximum. Thus, a "maxamplitude" is returned for every single maximum. This column can be used for statistics (calculation of the average amplitude).
- **Since last beat [sec]** – Running time after the last contraction/maximum for every single frame. Counting starts at the very first frame and the value is reset to zero at every single maximum identified.
- **Since last beat absolute time [sec]** – The overall time between the very first frame and the very first maximum, and later between two subsequent maxima printed at the current maximum; can be used for column statistics (**Fig. 15**).
- **Sum of beats** – Number of completed beats until the current moment at every identified maxima. Between the maxima, no value is returned.

- **Threshold (user defined, 10%, 50%, and 90%) [a.u.]** – returns the current absolute value of the threshold defined by the user and of the three constant ones, at 10%, 50% and 90%. This value is dynamic and recalculated for every single contraction depending on the difference between the current minimum and the next maximum (see "Maxamplitudes"), allowing robust compensation if the amplitude is shifted between the first and the last contraction(s) of the video, maybe due to a changing illumination or movement of the cell in x-y-direction. As well as the user defined threshold, the constant ones are also dynamically recalculated for every single maximum (see **Fig. 15** and **16** for details). If the amplitude exceed the according threshold (=local minimum + (according threshold in percent/100) x local amplitude), time measurement of both contraction time and systole begins. If the amplitude falls below the according threshold, counting of both diastole and contraction time ends. The results are presented in separate columns. While a constant threshold would only correctly intersect the local contraction that defined it, a dynamic threshold precisely adjusts the course of the amplitude, even if it becomes significantly shifted over time (**Fig. 17**).
- **Peak time (user defined, 10%, 50%, and 90%) (absolute) [sec]** – Returns the running peak time for every single frame the amplitude exceeds the according threshold. The **absolute peak time** shows only the overall time of the whole contraction according to the individual threshold and can be used for column statistics (**Fig. 15**).
- **Systolic interval (user defined, 10%, 50%, and 90%) (absolute) [sec]** – Returns the individual running times that start when the amplitude exceeds the according threshold and ends when the amplitude reaches the next maximum. The **absolute systolic interval** returns only the systolic overall time for every single threshold at the current maximum, while no value is given between the maxima (**Fig. 15**).
- **Diastolic interval (user defined, 10%, 50%, and 90%) (absolute) [sec]** – Here, a running time is returned that starts at the maximum of the current contraction and ends when the amplitude falls below the according threshold. **Diastolic interval absolute** returns only the overall time for every single threshold at the end of the systole. The sum of systolic and diastolic overall time for every single threshold equals the according absolute peak time (**Fig. 15**).

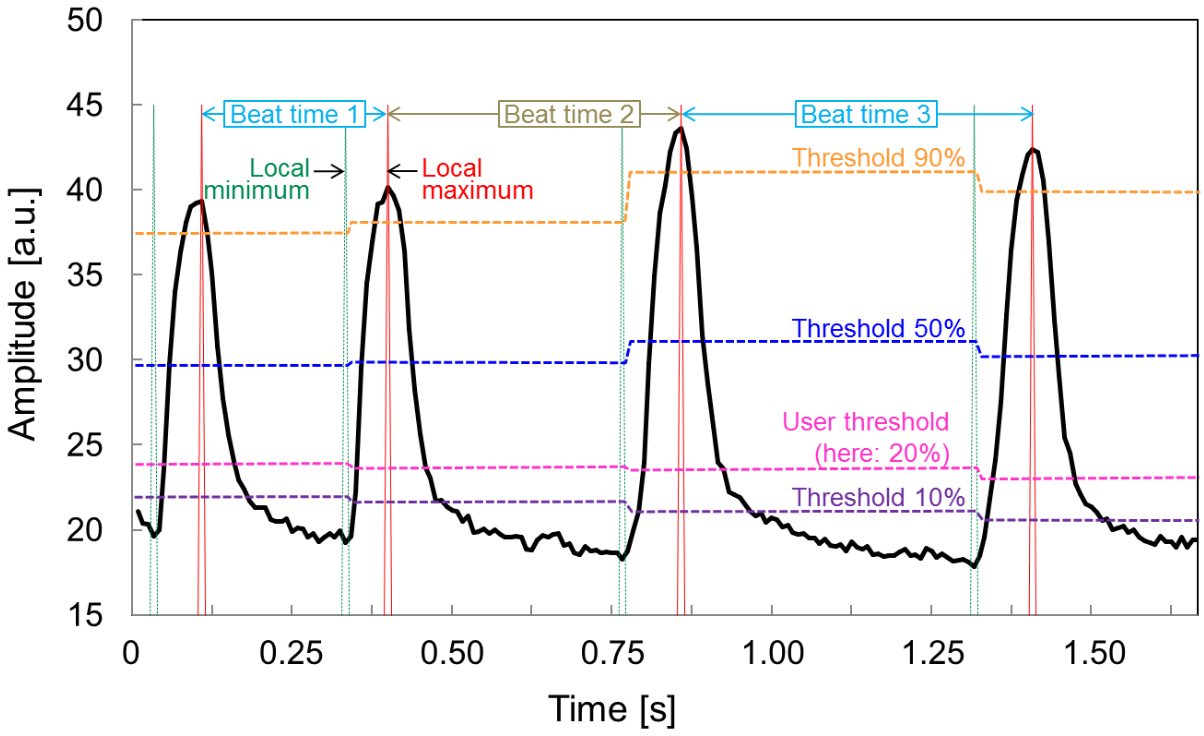

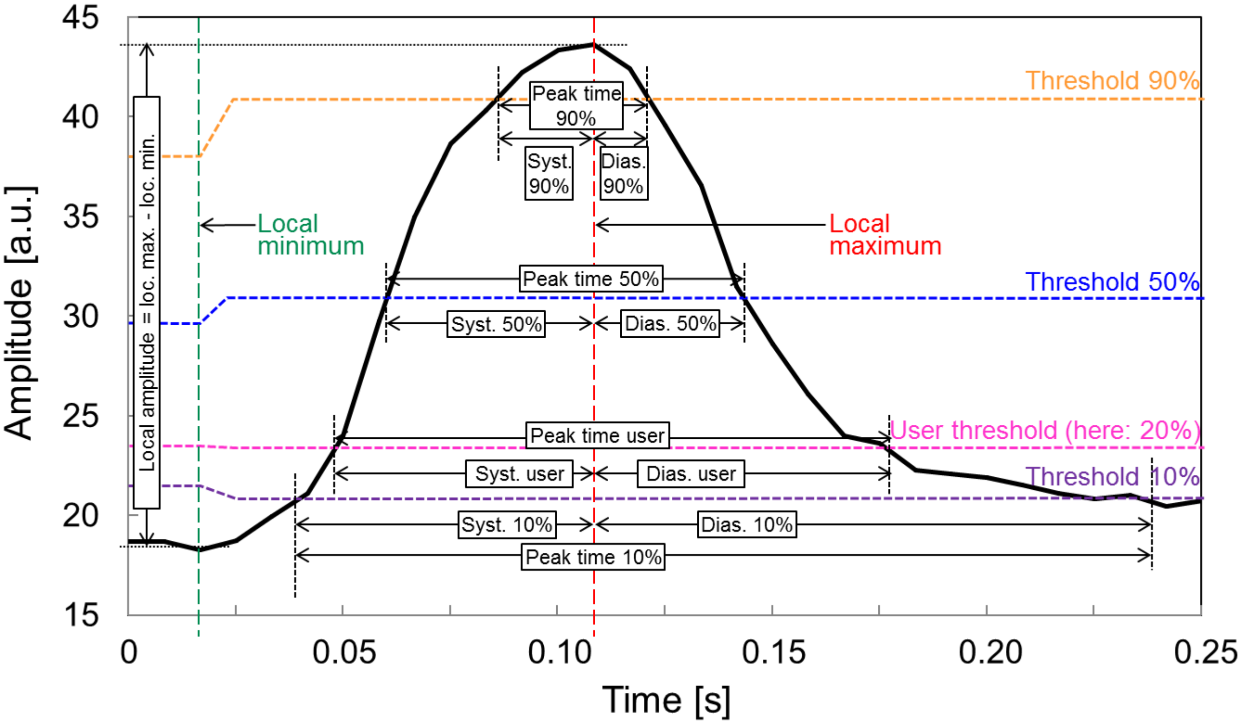

Fig. 15: The data extracted from a video file

This figure represents in its **upper panel** some of the data that are extracted by MYOOCYTER during evaluation of a video file: the times between two subsequent maxima (here: "Beat time 1/2/3"), all detected local maxima (red vertical spikes) and minima (green vertical spikes), the amplitude itself (black line), as well as specific information for four different thresholds (at 10, 20, 50 and 90% of the according local maximum).

The lower **panel shows** additional details of the parameters extracted: for every single contraction, the local minimum (green vertical dashed line) maximum (red vertical dashed line) is calculated, and also the four thresholds are readjusted to match the local amplitude (the difference between the local maximum and minimum, depicted on the left). A threshold of X% represents the "local minimum + X% of the local amplitude" and for every applied threshold the according systolic, diastolic and overall contraction times are calculated.

At the end of this table, also basic statistics and further information about the settings of the macro are added. Minimum, maximum, mean and standard deviation (StdDev) are given for beat times [sec], (Max)amplitudes [a.u.], peak times [sec], systoles [sec], and diastoles [sec] for all thresholds. Furthermore, several ratios like systole/diastole, amplitude/systole, amplitude/diastole, and amplitude/peaktime are calculated.

This statistics come in two different versions: the first one includes all detected contractions, the second one excludes both the first and the last "event", since it is highly probable, that the video does not start and end exactly in the resting phase between two contractions. Removing the first and the last "event" may improve statistics, especially for short videos containing only a few contractions.

Besides of the text file containing the results, there is also a text file named as "Amplitudes only" followed by date and time of the evaluation. This file is important for further processing of the data by the third available option ("**3. Re-Evaluation**") of MYOCYTER (**for details, please see section "*Further processing of the numerical output*"**).

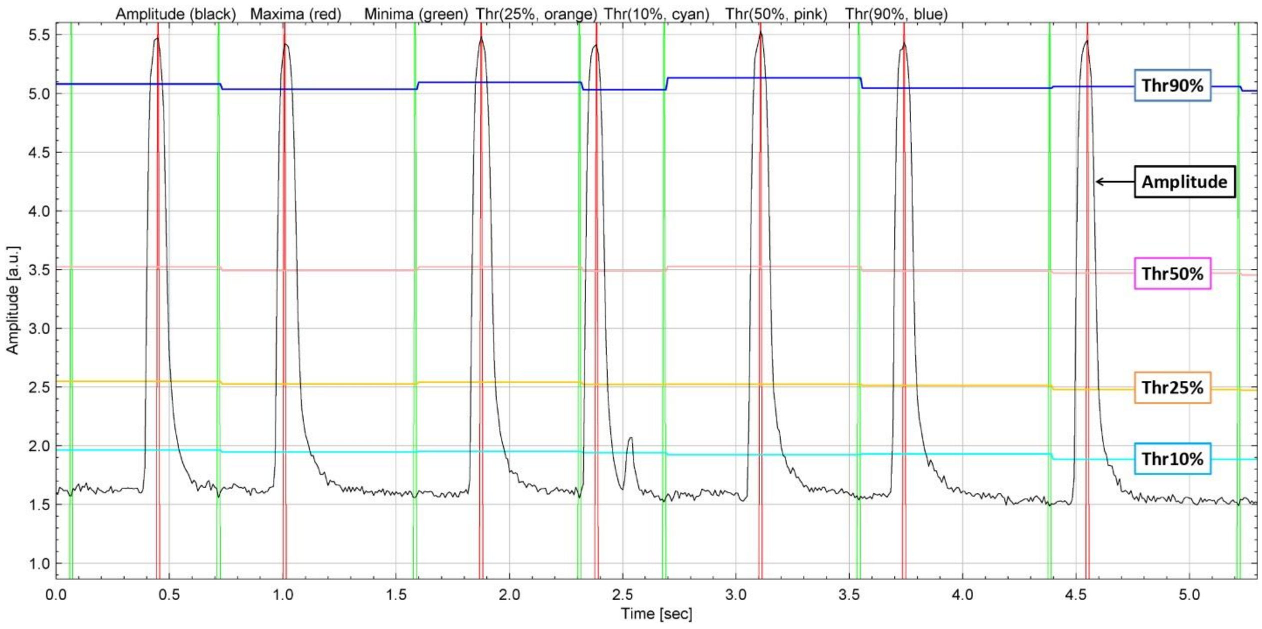

Fig. 16: The four dynamic thresholds

MYOCYTER automatically sets three different thresholds: 10% (cyan line), 50% (pink line), and 90% (blue line), while a fourth can be user-defined (in this case, 25% (orange line)). The black curve represents the amplitude, the vertical lines indicate detected maxima (red) and minima (green). The respective thresholds are calculated individually for each individual peak according to its maximum and the previous minimum: the difference between these two values is used to indicate exactly when the amplitude exceeds 10/25/50/90% of this altitude. For example, the threshold of 10% is calculated using the formula Thr(10%) = local minimum + 0.1*(local Maximum - local minimum), the other thresholds are calculated accordingly.

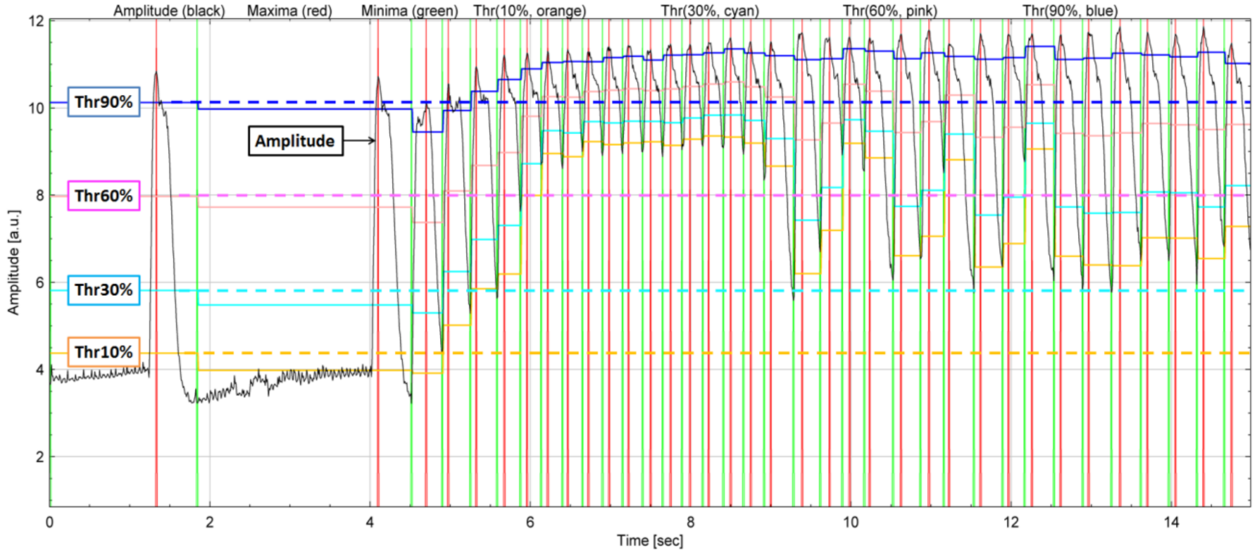

Fig. 17: Dynamic and static thresholding compared

In extreme, absolutely non-uniform cellular contractions, such as an arrhythmia, the heights of the amplitudes can be very different from each other. By means of dynamic thresholding, however, it is possible to adjust the thresholds locally to each individual maximum, while constant thresholds that are defined at the beginning of the measurement, would very quickly completely leave the amplitude. This figure shows the progression of initially

defined constant versus dynamically adjusting thresholds. Thr10% (orange), Thr30% (cyan), Thr60% (pink) and Thr90% (blue) are displayed both dynamically (solid lines) and constantly (dashed lines in the according color). After a short time, the constant Thr10% and Thr30% completely leave the amplitude (black curve), while all dynamic thresholds locally adapt from contraction to contraction and thus, can even precisely track "special cases" as in this example. The vertical lines represent recognized maxima (red) and minima (green). The numeric values of the local thresholds are determined by MYOCYTER based on the difference between a minimum and the following maximum.

The graphical output of MYOCYTER

In addition to the numerical, there is also a graphical output, which allows quick overview of both data and evaluation. Again, there is a subfolder called "dataplots". This subfolder also contains the numerical results as well as several graphical representations. The data of the amplitude are plotted together with all found maxima and minima (**Fig. 18**).

Besides of this, also every single identified object/cell is also stored in a separate video file. These files may be useful, if in some cases forcing of a reference frame should be necessary or if there is need to have only a single cell per video.

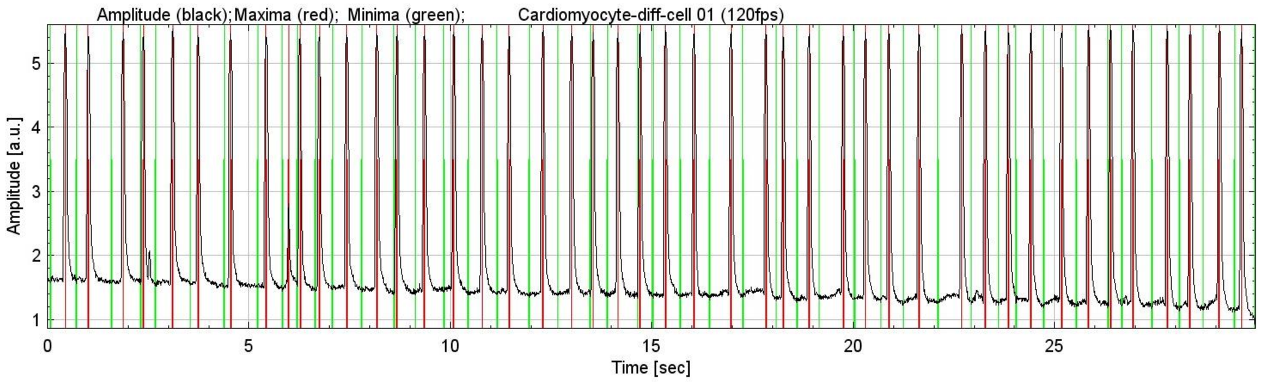

Fig. 18: Plot of the amplitude including all detected maxima and minima
The values of the amplitude (black curve) are plotted against time and presented with all detected maxima (red vertical lines) and minima (green vertical lines). The name of the evaluated video is found in the header of the graph (in this case "Cardiomyocyte"), as well as the corresponding identified cell (in this case "cell 01"). The indication "(120fps)" behind it stands for the automatically detected frame rate of the video. This representation is saved in two different resolutions (a small one for presentation in PowerPoint and a large one for a more detailed viewing).

Plots of the amplitudes of each detected structure (in two different resolutions) can be found there, as well as the animated amplitude-renderings, if the option "**Video output**" has been activated.

"**Video output**" creates an animation of the amplitude that is synchronized with the according cell. This feature is especially suitable for quick visual control of sample analysis as well as for data presentation (the MJPEG-video format of ImageJ is compatible with PowerPoint). How such a video looks is shown in **Fig. 19**. If the video of the identified cell is rather wide than high, it will be rotated 90 degrees to reduce the video size of the output (see **Fig. 19**, upper left corner). The frame rate of this video is 25 frames per second.

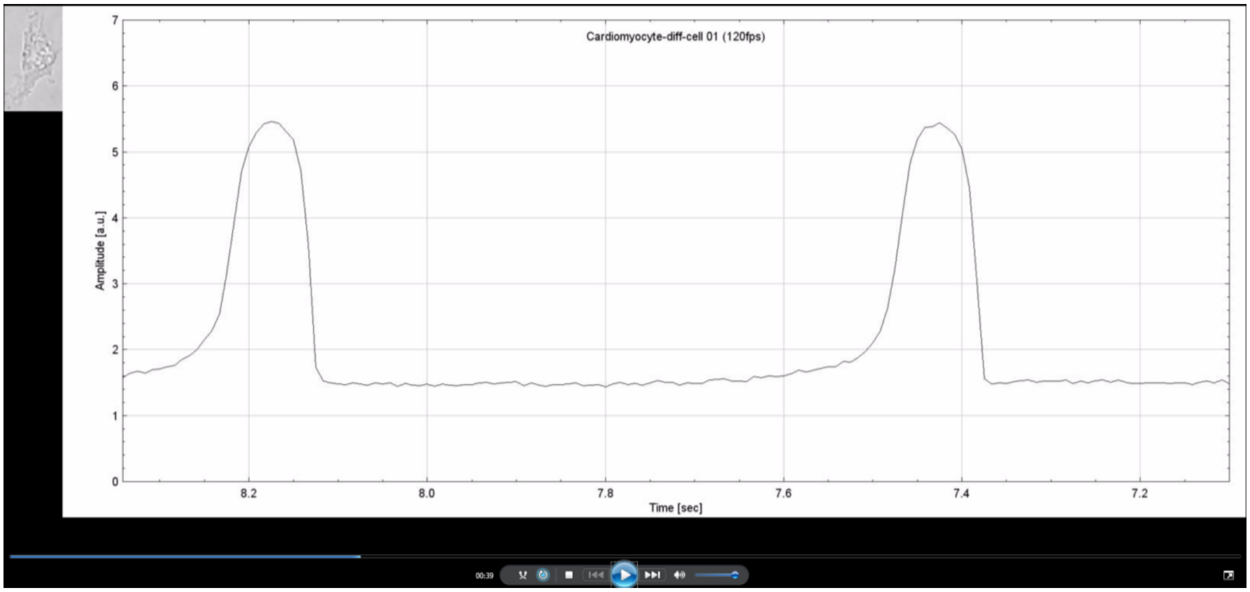

Fig. 19: The animated amplitude synchronized with the according cell
When the option "**Video output**" is activated, MYOCYTER outputs a high-resolution video that animates the amplitude (plotted against time) of the detected cell or structure together with it (small picture on the top left). The direction of movement of the animation runs from left to right. In this way, the contractions of the cell can be compared with the amplitude synchronized.

Further processing of the numerical output

The further data processing of the results is accomplished with the file "Amplitudes only" from the subfolder "diffMov/dataplots" of the evaluation folder. This requires the third option available in MYOCYTER ("**3. Re-Evaluation**").

The data of the amplitudes are provided as arrays in the form “amplitudes=newArray(y_1, y_2, …, y_n);” that is readable for ImageJ in macros. Those data can be directly opened by MYOCYTER for (re)processing of the amplitudes, enabling application of even 4 user-defined thresholds [%], an arbitrary value for “Detection” [a.u.] and also a different frame rate [1/sec], both calculating and plotting the new results in real time.

This allows fine-tuning of the applied settings in real time, without having to re-evaluate the video(s) of the cells.

How to import the data

Start MYOCYTER and select the third available option “**3. Re-Evaluation**” (see **Fig. 20**)

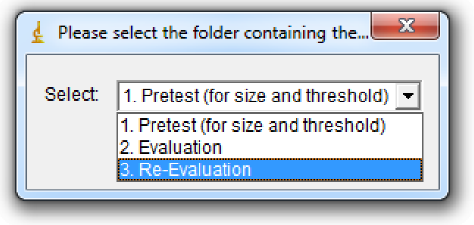

Fig. 20: 3. Re-Evaluation

Now, you are asked to select the according “Amplitudes only.txt” file of the evaluation you want to apply changed settings to (see **Fig. 21**). This file should be found in the subfolder “VidsMovement” > ”diffMov” > “dataplots” of any evaluation done with MYOCYTER.

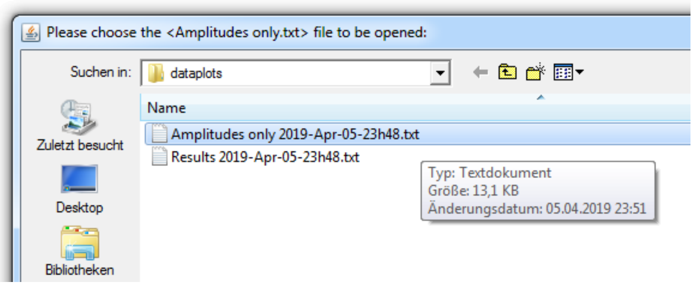

Fig. 21: Select the “Amplitudes only”-file.

After import of your data, you will be asked to apply several settings for “Detection”, 4 different thresholds (in percent), and also for the frame rate. (see **Fig. 22**). Higher values for “Detection” will result in the recognition of smaller peaks as whole contractions, as explained in detail in the following section.

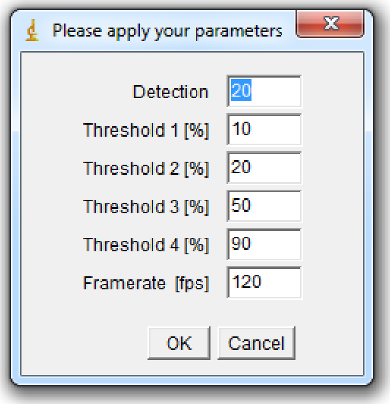

Fig. 22: Apply changed settings for re-evaluation of your data.

Detection:

As already mentioned, if this value is increased, even smaller changes in the amplitude are recognized as an independent peak. **Fig. 23** shows the effect of "**Detection**" on the identification of local maxima and minima.

Fig. 23, Panel A

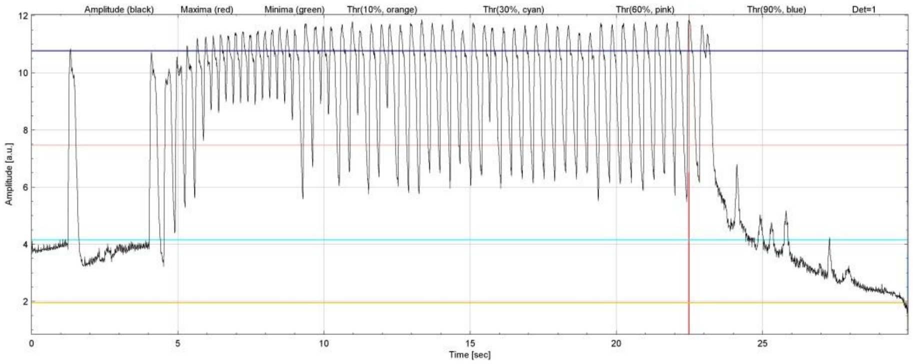

Fig. 23, Panel B

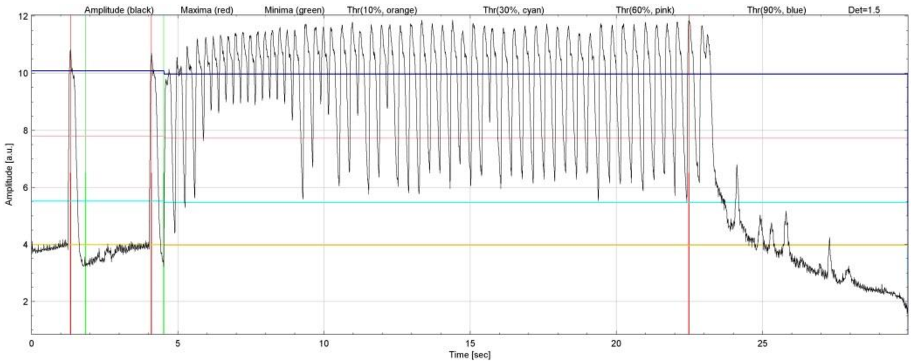

Fig. 23, Panel C

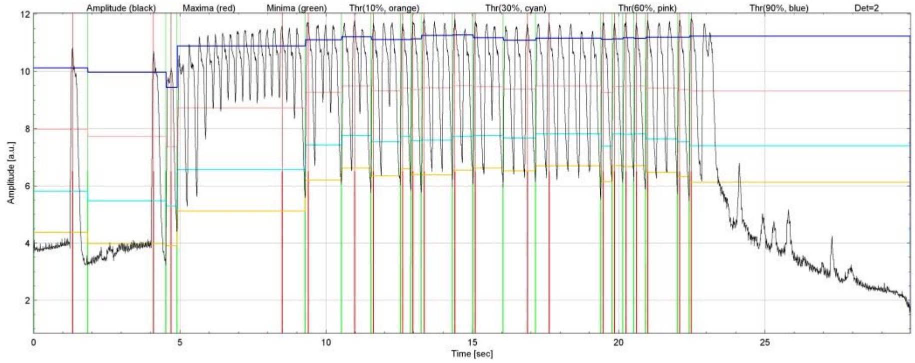

Fig. 23, Panel D

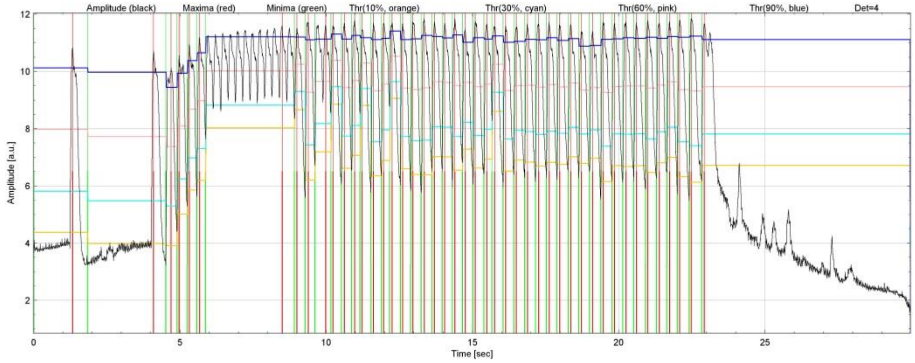

Fig. 23, Panel E

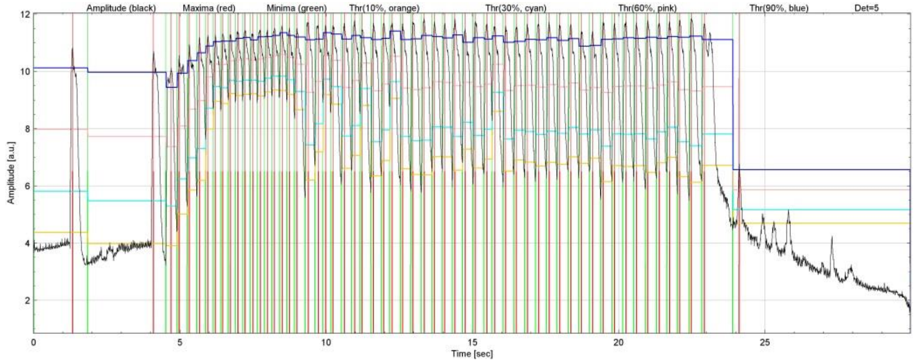

Fig. 23, Panel F

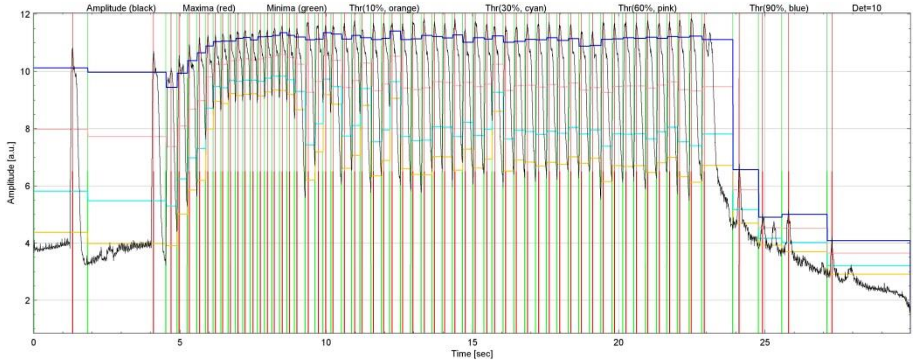

Fig. 23, Panel G

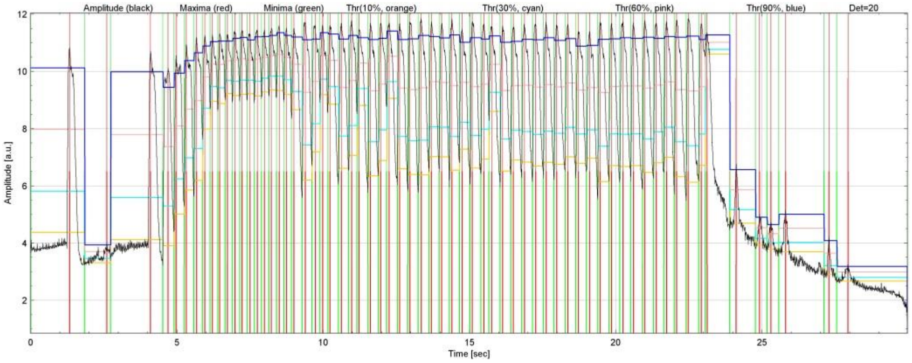

Fig. 23, Panel H

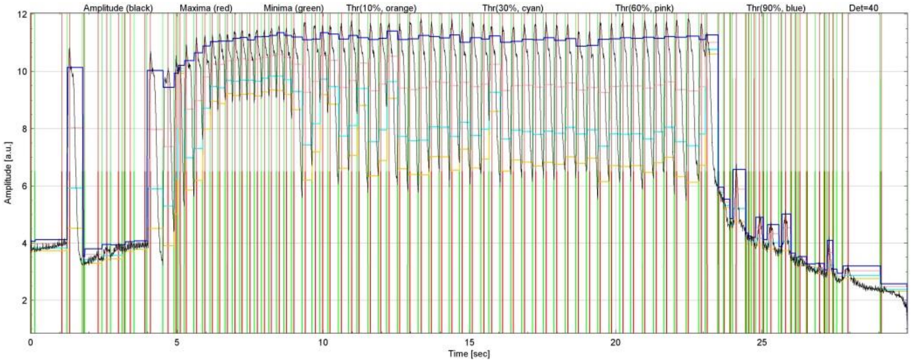

Fig. 23: The effects of the "Detection" value

The single parts of this figure (panel A-H) show the effect of increasing the "Detection" value on the recognition of maxima (red vertical lines) and minima (green vertical lines). The corresponding value used for detection can be found in the upper right corner of the respective panel that is original output of the "*MYOCYTER further data processing*" macro. Increasing this value increases also sensitivity in the detection of maxima and minima. If the value for detection set in the initial evaluation, which in our experience is between 5 and 10 in cell culture (in vitro cardiomyocytes), is set too high or too low, this can easily be corrected in further data analysis. If Detection is set too high (**Panel F-H**), ever smaller deviations of the amplitude (black curve) are recognized as independent maxima. The thresholds (10% as orange line, 30% cyan, 60% pink, 90% blue) automatically adjust to the detected maxima and minima, the changed times for systole, diastole, total peak time and distance between two contractions are calculated automatically. The values used for "Detection" are 1 (**panel A**), 1.5 (**panel B**), 2 (**panel C**), 4 (**panel D**), 5 (**panel E**), 10 (**panel F**), 20 (**panel G**), and 40 (**panel H**).

Threshold 1-4:

These values can be set arbitrarily by the user. As in the evaluation of a video, a threshold of x% defines x% of the difference between the local minimum and the local maximum of every single recognized contraction. The thresholds equal the setting "**[%] of max recognized as beat**" that can be applied in video evaluation (see section "**Processing your videos in MYOCYTER – Evaluation**" for details).

Frame rate:
This is the frame rate of the video in frames per second. It is necessary to define this value correctly so that the information on the timeline is correct. The frame rate of the corresponding video can be found (among others) in the “Results” file.

After defining the settings, the user will be asked to select the cell to be re-revaluated via a pull down menu (see **Fig. 24**). The typical nomenclature here is: “name of the video” + “recognized cell”. The according identified cells can be found as image file(s) in the main folder of the evaluation (see **Fig. 14**). Select the according cell and click >OK< to start the re-evaluation.

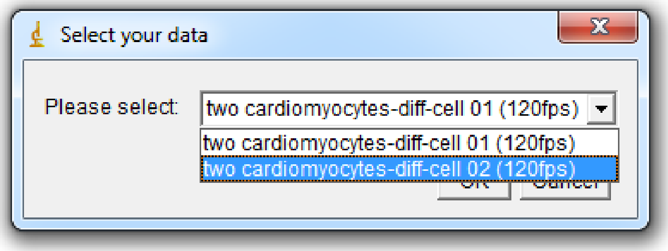

Fig. 24: Select a cell for re-revaluation.

The data output consists of a table containing all extracted parameters (see section “**The numeric output of MYOCYTER**”) and an according graphical output (see **Fig. 25**).

How to adjust the settings

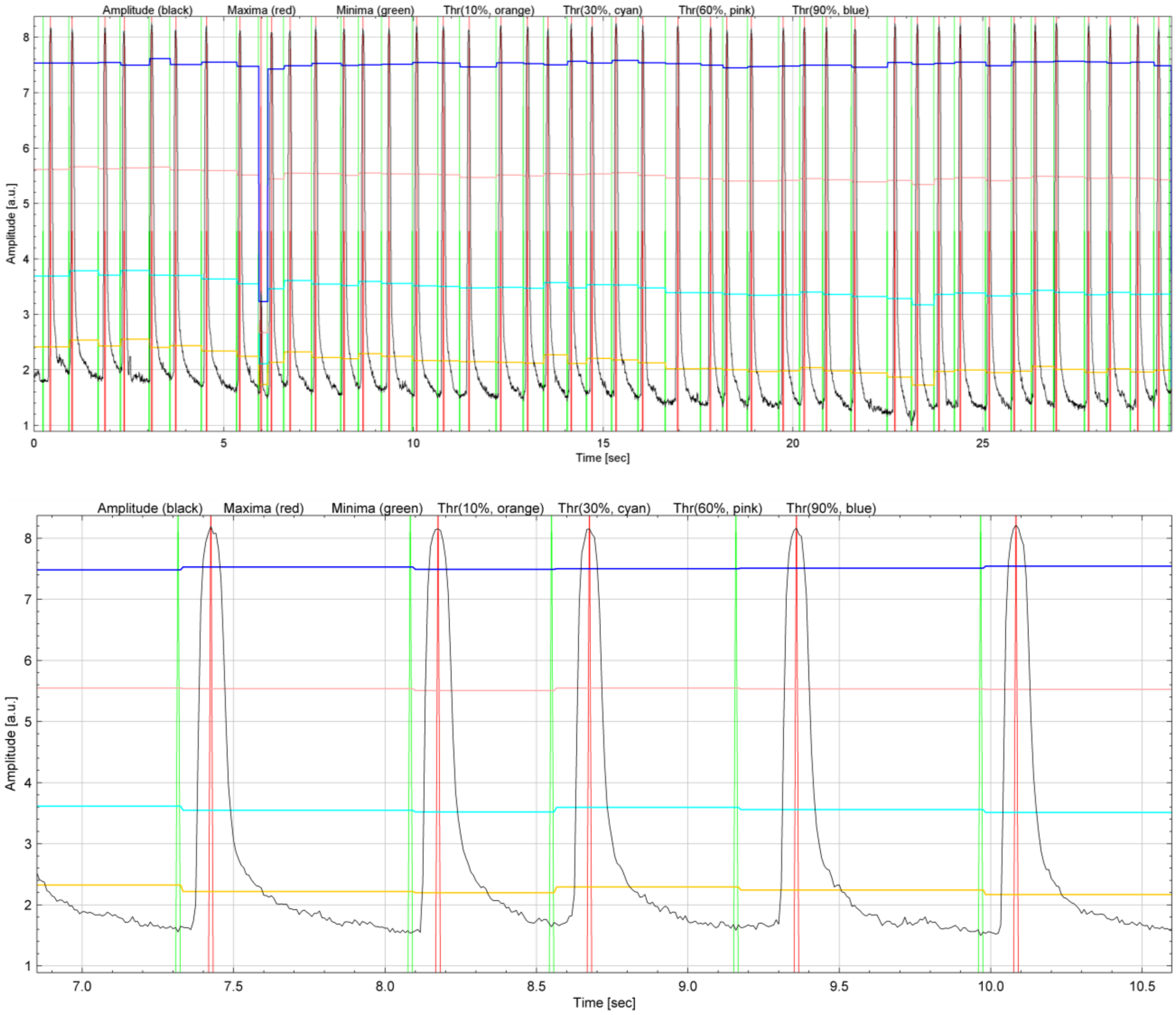

Fig. 25: Real time output of further data processing
The upper part of this figure shows the representation of the data in real time. The amplitude (black curve), each detected maximum (red vertical spikes), the detected minima (green vertical spikes) and the four freely definable thresholds are shown: in this case at 10% (orange), 30% (cyan), 60% (pink) and 90% (blue). These four thresholds can be changed arbitrarily. The lower part of the figure shows an enlarged section of the upper part for better visualization.

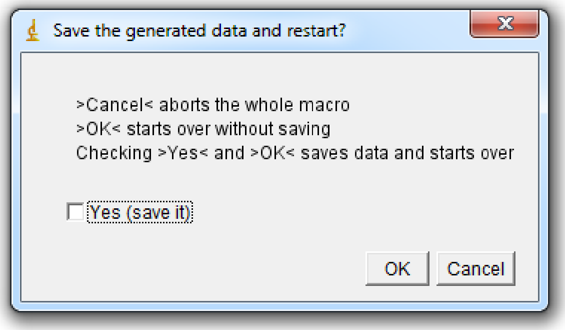

Fig. 26: Restart the re-evaluation
If you check the option “Yes (save it)”, both data table and graphical output are saved in the folder that already contains the re-evaluated “Amplitudes only.txt” after clicking >OK<. Just clicking >OK< without checking the option “Yes (save it)” restarts the evaluation process. Selection of >Cancel< aborts the whole macro.

If you restart the re-evaluation (**Fig. 26**) all generated data are deleted and you return to the mask for the new settings (**Fig. 22**). All parameters you applied in the last cycle of the re-evaluation are still there, in order to minimize the re-entry.

Interpreting the data

The most obvious parameters of the evaluation are amplitude, speed of movement (“Speed”), distance between two amplitudes (maxima), as well as systolic, diastolic and overall peak time of the four different thresholds.

Amplitude is given in arbitrary units and should be interpret carefully. Comparing different amplitudes makes (more) sense, especially if one observes the same cell before, during and after a certain “treatment”. In this case one can compare the amplitude after the treatment directly with a reference quantity, namely the amplitude before the treatment. Otherwise, the amplitude may be used with caution, because it can be very different from cell to cell even within the same sample (cultured under same conditions in the same dish).

Nevertheless, if the number of samples per group is sufficiently high and the objects look very uniform and of similar “transparency”, the amplitudes can still be statistically reliably compared with one another. Note, however, effects such as “granularity”: cells that contain more visible particles/structures than cells that are completely transparent will show more “change” from frame to frame even with the same contraction strength. Thus, higher values for both speed and amplitude will result. Treatments that increase or reduce “granularity” or “transparency” may consequently influence the according results for both amplitude and speed. In such cases the absolute parameters (distance between two amplitudes, systolic, diastolic and overall peak time given that are returned in seconds) may be the parameters of choice for further statistics.

This should be taken into account is different samples are compared.

Adapt the code

MYOCYTER is composed of individual sections with clearly defined subtasks. The corresponding comments in the format “// ** Comment **” are helpful if you want to quickly find your way around the code. If you want to change the code, change it step by step, check the results of the changes, and save each modified working version separately. In this way you save time if something does not work (correctly) by simply returning to the last known function.

If you change many things at the same time, troubleshooting is much more complicated. Take notes of what effects new code sections have, where these sections start and where they end.

It is also possible to change only small parts of the code to test them separately by using already prepared data (amplitudes=newArray (1.863, 1.8816, [...], 1.6082, 1.6329);) without having them to be extracted from a video with each test run. If the new code section works as desired, it can be embedded in the existing whole code.

If you change the code in the windows editor, be sure to disable the line break. Corresponding code editors that represent code in different colors and show with curly brackets, where these are opened and closed (sometimes many pages of code later) are very helpful in this work.

Corresponding ImageJ- or Java-forums contain a lot of useful hints for the solution of defined small sub-problems in macro-programming.

You can customize the code to your own needs, but we ask that you cite MYOCYTER appropriately in your work.

Additional material

Included in the “MYOCYTER”-package available at www.scyrus.de.

- Video of a **moving bar** (MJPEG compressed)
- Video of a **single cardiomyocyte**
- Video of an **adult cardiomyocyte** (small)
- Video with **alternating back and white frames**
- Zipped video of a **known declining function** (uncompressed)
- Video of an **adult cardiomyocyte** (big)
- Video of a **known declining function** (MJPEG compressed)
- Video of an **exposed mouse heart**
- Video of **two cardiomyocytes** for testing of **multi-cell-recognition**
- Video of a **water flea heart**
- The ImageJ-macro **code of “MYOCYTER”**

A complete representative evaluation

A complete example of a representative evaluation using the video file "Two cardiomyocytes.avi". The pretest of this video results in the overview image shown in **Fig. 27**. According to the pre-test, the values 77 (for "Threshold") and 8104 (for "Size") were applied for analysis.

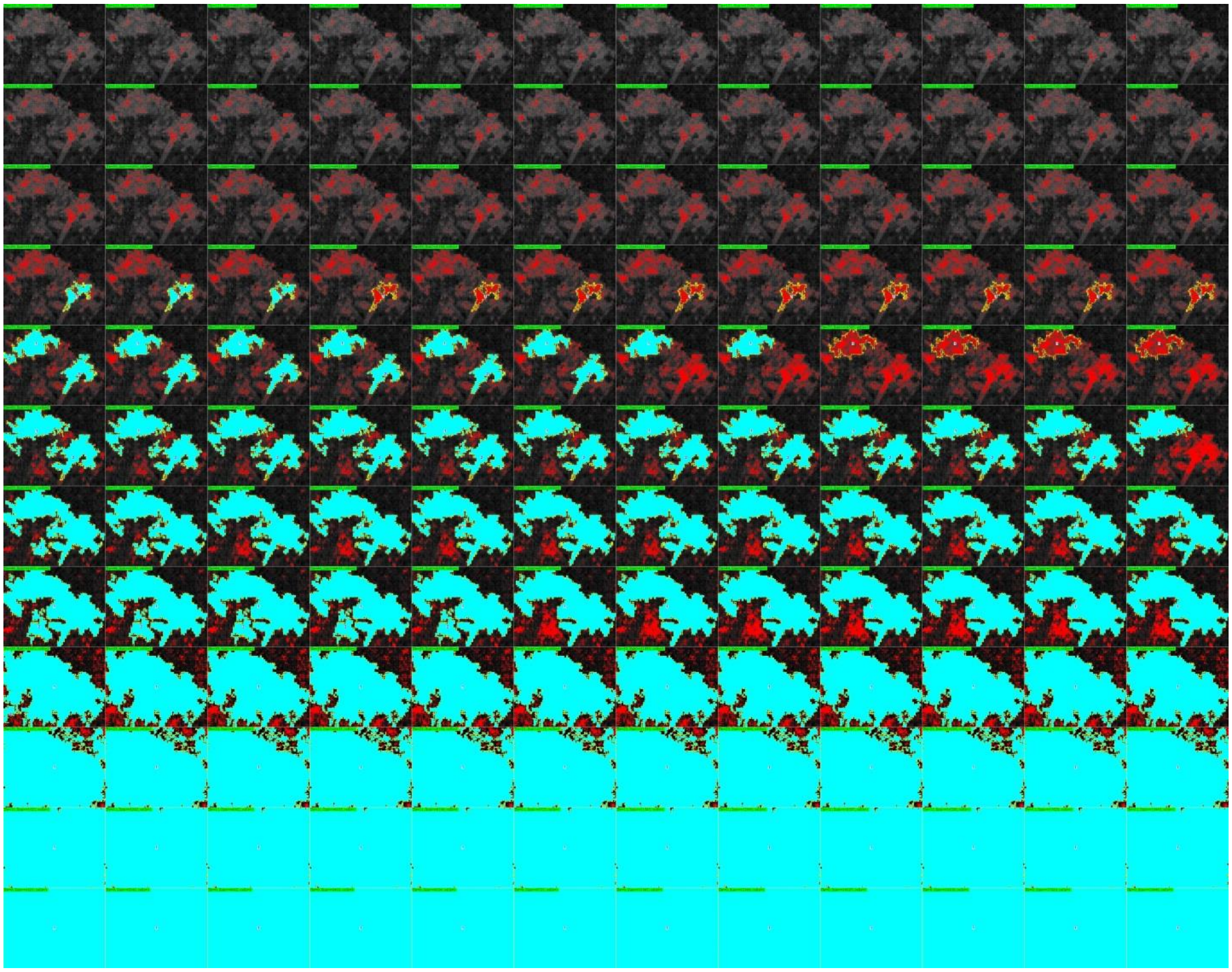

Fig. 27: Results of the pre-test ("LowMovement" applied) for the video "Two cardiomyocytes.avi"

MYOCYTER reliably detected two different cells (**Fig. 28**), which are evaluated independently. The reference images are calculated independently for each cell.

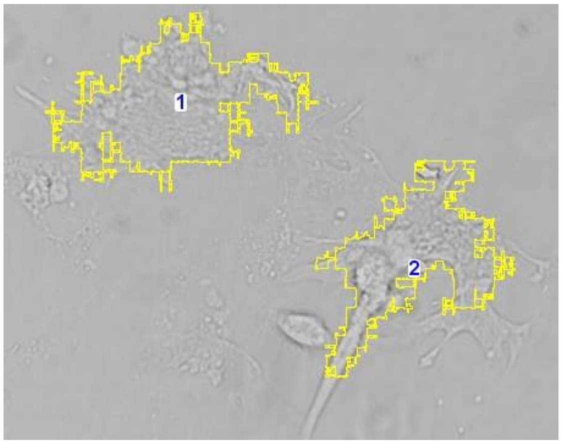

Fig. 28: The two recognized cells in the video file "Two cardiomyocytes.avi" after the settings 77 („Threshold") and 8104 („Size") according to the pretest shown in Fig. 22 were applied.

Both amplitudes of the identified cells are shown in **Fig. 29**: the upper panel represents "cell1", the lower panel "cell2". Besides of the values for threshold and size a "Detection" of 5 was applied (typical for cardiomyocytes).

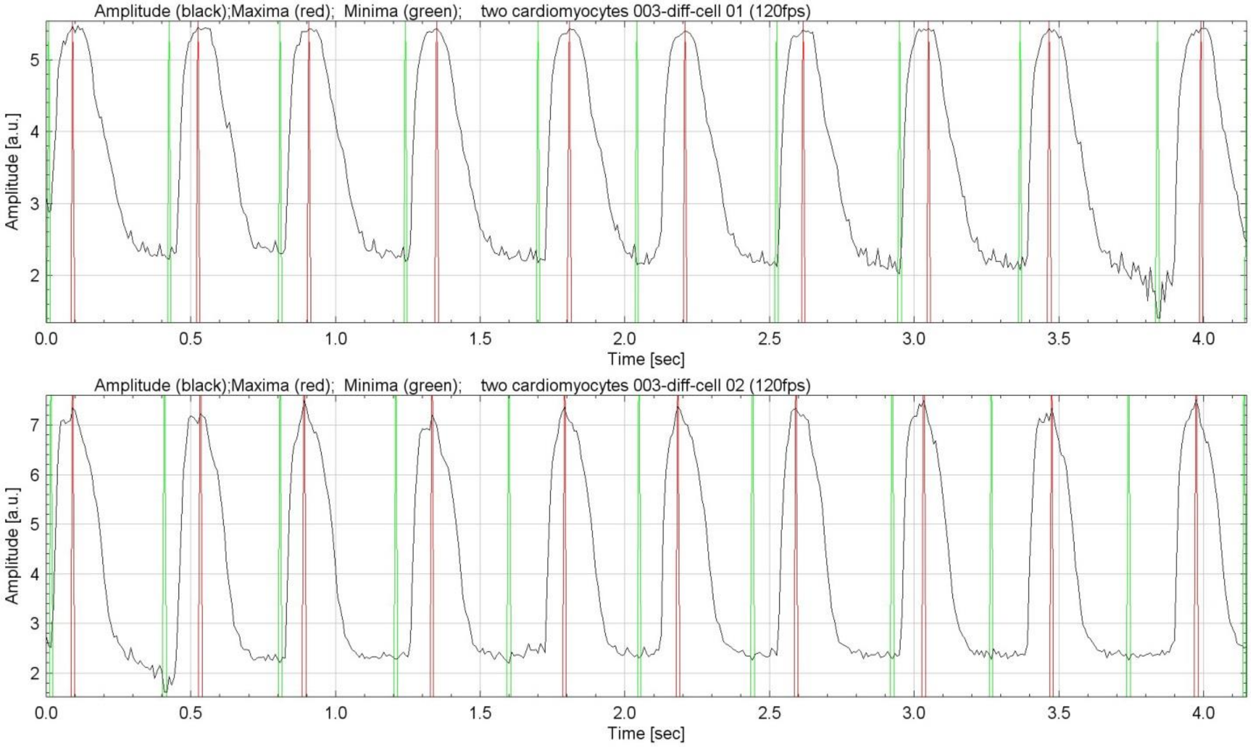

Fig. 29: The amplitudes plotted for the two recognized cells in the video file "Two cardiomyocytes.avi" The amplitudes are plotted in black, minima are represented as green, maxima as red spikes.

For "cell 1", 11 contractions were correctly recognized for "cell 2" 9 contractions. The statistical summary for both cells is summarized below:

Statistics fort he recognized „cell 1“:

Statistics INCLUDING the first and last event				
**Beats counted:**	**10**			
	**Min**	**Max**	**Mean**	**StdDev**
Beattimes [sec]	0.1	0.525	0.4	0.1124
Frequency [1/sec]	1.9048	10	3.0924	2.4347
Amplitudes [a,u,]	2.6002	4.0372	3.2842	0.3473
Peaktimes user [sec]	0.2083	0.2333	0.225	0.008333
Systoles user [sec]	0.06667	0.09167	0.07583	0.008287
Diastoles user [sec]	0.1417	0.1667	0.1509	0.008784
Peaktimes Thr10% [sec]	0.2167	0.2667	0.2509	0.01528
Systoles Thr10% [sec]	0.06667	0.1167	0.08083	0.01524
Diastoles Thr10% [sec]	0.15	0.1833	0.1741	0.01058
Peaktimes Thr50% [sec]	0.1583	0.1833	0.1722	0.007217
Systoles Thr50% [sec]	0.05	0.08333	0.0675	0.01072
Diastoles Thr50% [sec]	0.09167	0.125	0.1067	0.01024
Peaktimes Thr90% [sec]	0.075	0.1	0.09259	0.007733
Systoles Thr90% [sec]	0.01667	0.05833	0.04167	0.01179
Diastoles Thr90% [sec]	0.04167	0.06667	0.05167	0.007658
**Ratios:**				
Systole/diastole [user] mean:	0.5025			
Systole/diastole [Thr10%] mean:	0.4644			
Systole/diastole [Thr50%] mean:	0.6328			
Systole/diastole [Thr90%] mean:	0.8065			
Amplitude mean/systole [user] mean:	43.3083			
Amplitude mean/systole [Thr10%] mean:	40.6295			
Amplitude mean/systole [Thr50%] mean:	48.655			
Amplitude mean/systole [Thr90%] mean:	78.8212			
Amplitude mean/diastole [user] mean:	21.7604			
Amplitude mean/diastole [Thr10%] mean:	18.8668			
Amplitude mean/diastole [Thr50%] mean:	30.7895			
Amplitude mean/diastole [Thr90%] mean:	63.5655			
Amplitude mean/peaktime [user] mean:	14.5965			
Amplitude mean/peaktime [Thr10%] mean:	13.0884			

Amplitude mean/peaktime [Thr50%] mean:	19.0696			
Amplitude mean/peaktime [Thr90%] mean:	35.4695			
**Statistics WITHOUT the first and last event**				
**Beats counted:**	**8**			
	**Min**	**Max**	**Mean**	**StdDev**
Beattimes [sec]	0.3833	0.4583	0.4219	0.02437
Frequency [1/sec]	2.1818	2.6087	2.3774	0.1389
Amplitudes [a,u,]	3.1332	3.4119	3.2756	0.08468
Peaktimes user [sec]	0.2167	0.2333	0.2274	0.006299
Systoles user [sec]	0.06667	0.08333	0.075	0.006299
Diastoles user [sec]	0.1417	0.1667	0.1512	0.008909
Peaktimes Thr10% [sec]	0.25	0.2667	0.2571	0.007498
Systoles Thr10% [sec]	0.06667	0.09167	0.07812	0.008839
Diastoles Thr10% [sec]	0.1667	0.1833	0.1774	0.006299
Peaktimes Thr50% [sec]	0.1667	0.1833	0.1738	0.005751
Systoles Thr50% [sec]	0.05833	0.08333	0.06771	0.008259
Diastoles Thr50% [sec]	0.09167	0.125	0.1062	0.01157
Peaktimes Thr90% [sec]	0.09167	0.1	0.09524	0.004454
Systoles Thr90% [sec]	0.03333	0.05833	0.04375	0.008626
Diastoles Thr90% [sec]	0.04167	0.06667	0.05104	0.008259
**Ratios:**				
Systole/diastole [user] mean:	0.4969			
Systole/diastole [Thr10%] mean:	0.4404			
Systole/diastole [Thr50%] mean:	0.6373			
Systole/diastole [Thr90%] mean:	0.8571			
Amplitude mean/systole [user] mean:	43.6745			
Amplitude mean/systole [Thr10%] mean:	41.9276			
Amplitude mean/systole [Thr50%] mean:	48.378			
Amplitude mean/systole [Thr90%] mean:	74.8706			
Amplitude mean/diastole [user] mean:	21.6653			
Amplitude mean/diastole [Thr10%] mean:	18.4664			
Amplitude mean/diastole [Thr50%] mean:	30.8291			
Amplitude mean/diastole [Thr90%] mean:	64.1748			
Amplitude mean/peaktime [user] mean:	14.4057			

Amplitude mean/peaktime [Thr10%] mean:	12.7384			
Amplitude mean/peaktime [Thr50%] mean:	18.8459			
Amplitude mean/peaktime [Thr90%] mean:	34.3937			
**Reference Frame:**	**462**			
**Framerate [1/s]:**	**120**			

Statistics fort he recognized „cell 2“:

**Statistics INCLUDING the first and last event**				
**Beats counted:**	**10**			
	**Min**	**Max**	**Mean**	**StdDev**
Beattimes [sec]	0.1	0.5	0.3983	0.1117
Frequency [1/sec]	2	10	3.1031	2.433
Amplitudes [a,u,]	4.8353	5.6136	5.1485	0.2155
Peaktimes user [sec]	0.175	0.2167	0.187	0.01258
Systoles user [sec]	0.05	0.075	0.0625	0.009001
Diastoles user [sec]	0.1083	0.1417	0.1242	0.01142
Peaktimes Thr10% [sec]	0.2	0.3583	0.2222	0.0512
Systoles Thr10% [sec]	0.05	0.08333	0.06583	0.009977
Diastoles Thr10% [sec]	0.125	0.2833	0.155	0.04618
Peaktimes Thr50% [sec]	0.1417	0.1583	0.1509	0.006514
Systoles Thr50% [sec]	0.04167	0.06667	0.05667	0.008607
Diastoles Thr50% [sec]	0.075	0.1083	0.09333	0.01024
Peaktimes Thr90% [sec]	0.008333	0.075	0.05833	0.02187
Systoles Thr90% [sec]	0.008333	0.05	0.03083	0.01472
Diastoles Thr90% [sec]	0.01667	0.05	0.0325	0.01072
**Ratios:**				
Systole/diastole [user] mean:	0.5034			
Systole/diastole [Thr10%] mean:	0.4247			
Systole/diastole [Thr50%] mean:	0.6071			
Systole/diastole [Thr90%] mean:	0.9487			
Amplitude mean/systole [user] mean:	82.3758			
Amplitude mean/systole [Thr10%] mean:	78.2048			
Amplitude mean/systole [Thr50%] mean:	90.8556			
Amplitude mean/systole [Thr90%] mean:	166.9779			

Amplitude mean/diastole [user] mean:	41.4643			
Amplitude mean/diastole [Thr10%] mean:	33.216			
Amplitude mean/diastole [Thr50%] mean:	55.1623			
Amplitude mean/diastole [Thr90%] mean:	158.4149			
Amplitude mean/peaktime [user] mean:	27.5266			
Amplitude mean/peaktime [Thr10%] mean:	23.1682			
Amplitude mean/peaktime [Thr50%] mean:	34.1127			
Amplitude mean/peaktime [Thr90%] mean:	88.2597			
**Statistics WITHOUT the first and last event**				
**Beats counted:**	**8**			
	**Min**	**Max**	**Mean**	**StdDev**
Beattimes [sec]	0.3583	0.4583	0.4229	0.03385
Frequency [1/sec]	2.1818	2.7907	2.3789	0.2058
Amplitudes [a,u,]	4.9264	5.6136	5.1752	0.2085
Peaktimes user [sec]	0.175	0.2167	0.1881	0.01432
Systoles user [sec]	0.05	0.075	0.0625	0.00996
Diastoles user [sec]	0.1083	0.1417	0.125	0.0126
Peaktimes Thr10% [sec]	0.2	0.3583	0.2286	0.0573
Systoles Thr10% [sec]	0.05	0.08333	0.06563	0.0113
Diastoles Thr10% [sec]	0.125	0.2833	0.1604	0.05074
Peaktimes Thr50% [sec]	0.1417	0.1583	0.1512	0.005751
Systoles Thr50% [sec]	0.04167	0.06667	0.05625	0.009708
Diastoles Thr50% [sec]	0.075	0.1083	0.09375	0.01068
Peaktimes Thr90% [sec]	0.008333	0.075	0.05417	0.02271
Systoles Thr90% [sec]	0.008333	0.05	0.02917	0.01606
Diastoles Thr90% [sec]	0.025	0.05	0.03437	0.01039
**Ratios:**				
Systole/diastole [user] mean:	0.5034			
Systole/diastole [Thr10%] mean:	0.4091			
Systole/diastole [Thr50%] mean:	0.6			
Systole/diastole [Thr90%] mean:	0.8485			
Amplitude mean/systole [user] mean:	82.803			
Amplitude mean/systole [Thr10%] mean:	78.86			
Amplitude mean/systole [Thr50%] mean:	92.0034			

Amplitude mean/systole [Thr90%] mean:	177.435			
Amplitude mean/diastole [user] mean:	41.4015			
Amplitude mean/diastole [Thr10%] mean:	32.2609			
Amplitude mean/diastole [Thr50%] mean:	55.202			
Amplitude mean/diastole [Thr90%] mean:	150.5509			
Amplitude mean/peaktime [user] mean:	27.5137			
Amplitude mean/peaktime [Thr10%] mean:	22.6415			
Amplitude mean/peaktime [Thr50%] mean:	34.2296			
Amplitude mean/peaktime [Thr90%] mean:	95.5419			
**Reference Frame:**	**50**			
**Framerate [1/s]:**	**120**			
***** **Settings** *****				
Lower threshold:	**77**			
Cellsize:	**8104**			
[%] of Max recognized as beat:	**20**			
Detection:	**5**			
ImageJ-version:	**2,0,0-rc-69/1,52p**			
NO smoothing applied				
Evaluation date and time:	**2019-Jul-12-16h11**			

If for some reason this evaluation is not correct, the applied parameters can be easily changed in the post-processing of the numerical output. This requires the file "*amplitudes only.txt*" from the folder containing the results of this analysis, as described in detail in the section "**Further processing of the numerical output**".
